# Connectivity-based structural and functional parcellation of the human cortex using diffusion imaging and tractography

**DOI:** 10.3389/fnana.2012.00034

**Published:** 2012-08-29

**Authors:** Lauren L. Cloutman, Matthew A. Lambon Ralph

**Affiliations:** Neuroscience and Aphasia Research Unit, School of Psychological Sciences, University of ManchesterManchester, UK

**Keywords:** connectivity, cytoarchitecture, diffusion, functional specialization, tractography

## Abstract

The parcellation of the cortex via its anatomical properties has been an important research endeavor for over a century. To date, however, a universally accepted parcellation scheme for the human brain still remains elusive. In the current review, we explore the use of *in vivo* diffusion imaging and white matter tractography as a non-invasive method for the structural and functional parcellation of the human cerebral cortex, discussing the strengths and limitations of the current approaches. Cortical parcellation via white matter connectivity is based on the premise that, as connectional anatomy determines functional organization, it should be possible to segregate functionally-distinct cortical regions by identifying similarities and differences in connectivity profiles. Recent studies have provided initial evidence in support of the efficacy of this connectional parcellation methodology. Such investigations have identified distinct cortical subregions which correlate strongly with functional regions identified via fMRI and meta-analyses. Furthermore, a strong parallel between the cortical regions defined via tractographic and more traditional cytoarchitectonic parcellation methods has been observed. However, the degree of correspondence and relative functional importance of cytoarchitectonic- versus connectivity-derived parcellations still remains unclear. Diffusion tractography remains one of the only methods capable of visualizing the structural networks of the brain *in vivo*. As such, it is of vital importance to continue to improve the accuracy of the methodology and to extend its potential applications in the study of cognition in neurological health and disease.

## Introduction

For over 100 years, one of the most prominent and enduring research endeavors in neuroscience has involved the delineation and mapping of anatomically distinct subregions within the cerebral cortex (see Zilles and Amunts, 2010, for a historical review). The focus on the parcellation of the cortex via its anatomical properties has been based on the foundation that structure determines function and, as such, structurally-defined boundaries may be used to infer regions of functional specialization (Johansen-Berg et al., 2004; Behrens and Johansen-Berg, 2005; Anwander et al., 2007). Thus, elucidating the underlying structural organization of the brain is seen as of vital importance in understanding and mapping brain function. To date, however, no universally accepted parcellation scheme currently exists for the human brain (Toga et al., 2006; Perrin et al., 2008).

An important property of the neuronal populations which comprise the cerebral cortex is the observation that their organization and distribution is not random, and neurons with similar microstructural properties cluster together to form coherent regions (Toga et al., 2006; Knosche and Tittgemeyer, 2011). As a consequence, anatomical microstructure has become the principal marker used to define cortical boundaries in both humans and non-human primates (Amunts et al., 2007; Zilles and Amunts, 2010; Geyer et al., 2011). In his seminal work on cerebral localization, Brodmann (1909) conducted his now famous parcellation of the cerebral cortex based on cytoarchitectonics, differentiating cortical regions on the basis of variations in cellular lamination patterns. Since then, cytoarchitecture has become one of the most prominent neuroanatomical features used for defining the structural and functional organization of the brain, becoming the “gold standard” for cortical parcellation (see e.g., Mesulam and Mufson, 1982; Amunts et al., 1999; Schleicher et al., 1999; Kotter et al., 2001b; Morosan et al., 2001, 2005; Eickhoff et al., 2005b, 2006; Caspers et al., 2006; Gregoriou et al., 2006; Belmalih et al., 2009; Gallay et al., 2012). In addition, although less prevalent, a number of other microstructural elements have also been used to differentiate segregated cortical regions, including myelination patterns (i.e., myeloarchitecture; Walters et al., 2003; Annese et al., 2004; Eickhoff et al., 2005b; Belmalih et al., 2009; Glasser and Van Essen, 2011; Gallay et al., 2012), and receptor binding and (immuno)histochemical properties (i.e., receptoarchitecture/chemoarchitecture; Carmichael and Price, 1994; Hof and Morrison, 1995; Kotter et al., 2001b; Ongur et al., 2003; Geyer et al., 2005; Morosan et al., 2005; Belmalih et al., 2009; Gallay et al., 2012).

Cellular microstructure, however, is but one anatomical feature which may be used for the delineation of structural and functional boundaries in the cortex. Brain function is strongly constrained by both anatomical microstructure and connectivity—cytoarchitecture determines a region's local processing capabilities whilst its connectivity governs the nature and flow of information to and from an area (Hilgetag and Grant, 2000; Passingham et al., 2002; Johansen-Berg et al., 2004; Keidel et al., 2010). Importantly, higher cognitive functioning is not underpinned by isolated cortical areas but involves the interaction and integration of complex networks of widely distributed interconnected brain regions (Felleman and Van Essen, 1991; Sporns et al., 2005; Bullmore and Sporns, 2009; Rubinov and Sporns, 2010; Stam, 2010; Bullmore and Bassett, 2011). Indeed, a region's pattern of corticocortical connections has been viewed as one of the most important structural features with respect to determining function (Sur et al., 1988; Knosche and Tittgemeyer, 2011). To quote Marsel Mesulam (2006), “nothing defines the function of a neuron better than its connections.”

Due to the importance of neural connectivity in determining a region's functional repertoire, it has been postulated that brain regions performing different functions will require different connectional architectures and, as such, it should be possible to segregate functionally-distinct cortical regions by identifying similarities and differences in long-range connectivity patterns (Hilgetag and Grant, 2000; Johansen-Berg et al., 2004; Behrens and Johansen-Berg, 2005; Anwander et al., 2007; Klein et al., 2007; Perrin et al., 2008; Knosche and Tittgemeyer, 2011). Indeed, as was noted by Brodmann himself:

“… one can distinguish three forms of histological localization: *cytoarchitectonics*, *myeloarchitectonics*, and *fibrilloarchitectonics* …. The study of the fibrillary features of the cerebral cortex, or *fibrilloarchitectonics*, still remains in its infancy. Those studies of the local neurofibrillar structure of the central cortex that are available lead us to suppose that after the preliminary localization work is complete, a systematic study of these features might reveal topographic differences that remain more or less undetected with the other methods.”

Brodmann (1909, p. 4)

Recent years have seen an explosion of research on the parcellation of the cortex via white matter connectivity. A crucial impetus behind this shift has been the advent of modern diffusion neuroimaging and probabilistic tractography, capable of visualizing long-range white matter tracts *in vivo* (Conturo et al., 1999; Parker et al., 2002, 2003; Hagmann et al., 2006). To date, accurate visualization and identification of the brain's cellular composition and underlying microstructure has only been possible via post-mortem histological studies, limiting its utility in the study of functional specialization within the human brain. Consequently, researchers have attempted to use surrogate markers to identify the brain's underlying parcellation, such as gross sulcal/gyral landmarks. While such an approach may be successful for certain brain regions—most notably the primary and secondary cortical areas—for higher-order cortical regions, the brain's observable macroarchitecture is not a reliable indicator of cytoarchitectonic borders (Fischl et al., 2008). In contrast, recent methodological advances in the tractographic approach have enabled the delineation of white matter fiber pathways within the living brain, even in regions of high anatomical complexity (Parker and Alexander, 2005). The approach has increasingly been used to map the complex interconnected networks underlying higher brain function including uniquely human skills such as language (see e.g., Lehericy et al., 2004; Catani et al., 2005, 2007; Makris et al., 2005; Parker et al., 2005; Barrick et al., 2007; Catani and Thiebaut de Schotten, 2008; Frey et al., 2008; Glasser and Rilling, 2008; Saur et al., 2008, 2010; Makris and Pandya, 2009; Ford et al., 2010; Turken and Dronkers, 2011; Cloutman et al., 2012). Diffusion tractography has enabled the parcellation of the cortex into functionally specialized subregions via their unique complement of corticocortical connections *in vivo*. A plethora of studies have been conducted aimed at furthering the technique, improving its accuracy and exploring its applications in the study of the structural and functional organization of the brain in normal and patient populations. However, the functional validity of these tractographically-defined cortical regions and degree of correspondence and relative functional importance of cytoarchitectonic- versus connectivity-based parcellations still remains unclear.

In the current paper, we review the use of *in vivo* diffusion imaging and white matter tractography as a non-invasive method for the structural and functional parcellation of the human cerebral cortex. We first explore the relationship between cellular and connective architecture as it relates to the correlation between cytoarchitectonic- versus connectivity-derived parcellations, and the relative influence of these two neuroanatomical features in the determination of cortical function. We then provide a methodological overview of white matter tractography and connectivity-based parcellation approaches, discussing the strengths and limitations of the tractographic approach and its use as a technique for the delineation of structural and functional subdivisions within the human cortex. Existing evidence regarding the efficacy of this connectional parcellation technique is examined and the correspondence between tractographic and cytoarchitectonic parcellations is explored. Finally, we review alternative structural and functional parcellation methodologies, and their relationship with cytoarchitectonic and structural connectivity-based techniques.

## The relationship between connectivity, cytoarchitecture, and cortical function

Cytoarchitecture and connective architecture are complementary anatomical properties of the brain, which are both are heavily implicated in determining a region's functional capabilities. In order to reconcile the information provided by the two different structural features in relation to the functional organization of the cortex, it is of vital importance to understand the relationship between cellular organization and neural connectivity, and their relative influence in determining cortical function.

The neurons which compose the cortex have a range of physiological and structural properties which govern both a region's local computational processing capabilities and the organization of connective inputs and outputs (Jacobson and Marcus, 2008). Pyramidal cells comprise approximately two-thirds of neurons within the cortex. Axons from these cells form the majority of corticocortical connections, with small/medium sized cells terminating as association fascicles within the cortex, while large/giant cells form long-range association, callosal, and subcortical projection fibers. In terms of their electrophysiology, these neurons mainly demonstrate regular spiking, although some deeper layer cells may demonstrate burst firing (repetitive discharge) and form excitatory synaptic connections. The other principal cell type within the cortex are stellate cells, which are predominantly present in layer IV of the sensory cortex but are found in all laminar layers. These cells are associated with short axons which only project locally and do not participate in long-range connectivity. Electrophysiologically, these neurons are fast spiking with brief action potentials and form inhibitory synaptic connections.

An essential design principle of the cerebral cortex is the horizontal laminar arrangement of these cortical neurons, producing well-defined cellular layers (six within the neocortex, three within the allocortex), which vary in their composition across regions of the cortex (Brodmann, 1909). These laminar patterns have been found to have a strong systematic influence on the origins and terminations of neural connections across the cortex. Specifically, feedforward connective pathways arise predominantly from superficial laminar layers (II/III) and terminate in granular layer IV, while complementary feedback connections originate from deep cortical layers (V/VI) and terminate mainly in extragranular laminae outside layer IV (Rockland and Pandya, 1979; Van Essen and Maunsell, 1983; Felleman and Van Essen, 1991). Lateral (horizontal) connections demonstrate a more bilaminar pattern, with origins from both superficial and deep layers and terminations across all layers. Studies with non-human primates have identified that the principal determinant of this laminar regularity in the origin/termination of cortical connections is the relative cytoarchitectonic differentiation of the source and target cortical areas (Barbas, 1986; Barbas and Rempel-Clower, 1997; Rempel-Clower and Barbas, 2000; Medalla and Barbas, 2006; Hilgetag and Grant, 2010). Neuronal projections which arise from highly differentiated laminar regions (i.e., well defined layers, especially layer IV), which target less differentiated regions will principally originate from superficial laminar layers, while less-differentiated to more highly-differentiated projections will arise predominantly from deeper laminar layers. As such, not only is cytoarchitecture a strong determinant of the local processing capacity of a cortical region but it also has a strong influence on the organization of neural connectivity, governing and predicting origin/termination patterns of corticocortical connections.

While cytoarchitecture may influence the brain's connective architecture, there is strong evidence to indicate that the reverse is also true. Studies have revealed that neural connectivity is a key determinant of the anatomical and physiological features of a cortical region, strongly influencing both cytoarchitectural composition and neuronal response properties (for reviews, see Pallas, 2001; Ragsdale and Grove, 2001; Horng and Sur, 2006; Majewska and Sur, 2006). For example, a study conducted by Rakic et al. (1991), which explored the mechanisms underlying the formation of cytoarchitectural regions by reducing thalamic projections to the primate primary visual cortex (area 17) during a midgestational period, induced a subregion of area 17 to receive inputs normally destined for the secondary visual cortex (area 18). It was observed that the inappropriate input to this region resulted in the formation of a novel cytoarchitectonic region which had a cellular lamination pattern that was cytologically neither 17 nor 18 but a hybrid between the two. As such, while cortical regions appear to have a pre-programmed cytoarchitectonic composition (referred to as a protomap), the final cellular organization may be strongly influenced by the received inputs and their computational requirements.

There is also strong evidence to indicate that connective inputs have a profound influence in the determination of functional specificity within the cortex. In a series of elegant studies, Sur et al. experimentally induced retinal projections to innervate the medial geniculate nucleus (MGN) in the ferret brain, resulting in visual input to the auditory thalamus and primary auditory cortex (Sur et al., 1988; Roe et al., 1992; Angelucci et al., 1997; Sharma et al., 2000). This resulted in an alteration of the anatomical and physiological features of the MGN such that they more closely resembled that of the (visual) lateral geniculate nucleus, with the clustering of retinal projections and eye-selective cellular segregation (Angelucci et al., 1997). Unlike the study of Rakic et al. (1991), the cytoarchitectural properties of the “rewired” MGN retained its normal organization, suggesting that there may be some limitations to the influence of connective input on cellular lamination patterns. Importantly, the novel visual inputs had a strong effect on the functioning of neurons within the primary auditory cortex. Specifically, these rewired neurons were found to demonstrate visual response properties, responding to visual-field stimulation and displaying computational response characteristics normally observed in the primary visual cortex, namely orientation tuning and direction selectivity (Sur et al., 1988; Roe et al., 1992; Sharma et al., 2000). Thus, the functioning of neurons within a cortical region appears to be predominantly determined by their connective inputs, so much so that when sensory inputs are altered, cellular response features and functional properties develop to reflect the new domain rather than the old.

The preceding discussion has revealed a complex interplay between cortical microstructure and connective architecture in determining the anatomical and functional organization of the cortex. While cytoarchitecture determines a region's local processing capabilities, it may also strongly influence corticocortical connection patterns. Reciprocally, there is some evidence to indicate that neural connectivity may exert a strong influence on the anatomical and physiological characteristics of a cortical region. In relation to functional organization and specialization, a region's pattern of input and output connectivity determines both the nature of the information available to a region for processing and its capacity to transmit to and influence the processing of other cortical areas. As such, corticocortical connectivity is a strong determinant of a region's functional repertoire. There is strong evidence, however, that the influence of connectivity on cortical function may go well beyond this, such that a region's neural functioning may be ultimately determined by its connective inputs, at least at the local level. The importance of neural connectivity in determining functional specialization suggests that the mapping and parcellation of cortical regions on the basis of their connective architecture may provide crucial insights into the underlying functional organization of the cerebral cortex. Consequently, researchers have begun to explore the use of *in vivo* diffusion tractography and connectivity-based parcellations in the delineation of structural and functional subdivisions within the brain.

## Diffusion tractography and connectivity-based parcellation techniques

Connectivity-based parcellations may be conducted using any methodology capable of mapping the white matter pathways of the brain, specifically tracer injections and diffusion tractography. While tracer studies can provide extremely fine-grained information about white matter anatomy, such a methodology cannot be extended to the study of whole brain connectivity and, most obviously, cannot be used to study the living brain (Jones, 2010). As such, the principal methodology utilized in connectivity-based parcellations has been diffusion magnetic resonance imaging (MRI) and tractography. In the following section, we briefly outline the methodological underpinnings of diffusion MRI and white matter tractography techniques, before reviewing statistical approaches developed to parcellate the cortex based on the connectivity profiles identified.

### Methodological foundations of diffusion MRI and tractography

Diffusion refers to the random motion of water molecules (sometimes referred to as Brownian motion) that occurs as a result of intrinsic thermal energy (see Mori, 2007, and Johansen-Berg and Behrens, 2009, for a thorough treatment of the topic; see also Le Bihan, 2007, for a review of neuroanatomical basis of diffusion in the brain). Within a free medium, water molecules are able to move in all spatial directions, following a three-dimensional Gaussian distribution (isotropic diffusion). The neural architecture of the brain serves to hinder molecular movement and results in greater displacement along a specific direction (anisotropic diffusion) in regions with highly-ordered tissue structure, such as axon bundles. Specifically, within white matter, diffusion is less hindered parallel to the principal axis of a fiber bundle than perpendicular to it, meaning that the direction of greatest molecular displacement (principal diffusion direction) aligns well with the dominant fiber orientation. MRI pulse sequences can be modified to enhance sensitivity to this diffusion such that the signal becomes increasingly attenuated with the increasing displacement of water molecules (Stejskal and Tanner, 1965). When this displacement is measured along multiple directions, a measure of the degree and direction of diffusion can be obtained, enabling calculation of the degree of anisotropy and identification of the principal diffusion direction (often referred to as the principal eigenvector). The diffusion displacement distributions obtained may then be used to infer the organization of white matter and the reconstruction of their trajectories via tractography.

There are currently two main tractographic approaches for the mapping of neural fiber pathways: deterministic (Conturo et al., 1999; Jones et al., 1999; Mori et al., 1999; Basser et al., 2000) and probabilistic (Behrens et al., 2003b; Parker and Alexander, 2003, 2005; Parker et al., 2003). In deterministic tractography, the three-dimensional pattern of diffusion anisotropy is used to calculate a diffusion tensor model, which describes the direction (eigenvector) and length (eigenvalue) of diffusion along the three directional axes. During tract reconstruction, a single streamline is propagated from voxel to voxel along the line of the principal eigenvector of the diffusion tensor. There is a degree of error inherent in any estimation of the diffusion tensor within a given voxel, producing a level of uncertainty in the prediction of fiber orientation. Deterministic algorithms traditionally do not account for such errors and are unable to provide a measure of the uncertainty of the reconstructed pathways (Jones, 2008a, 2010). In addition, in regions of complex fiber architecture (as in the case of voxels containing more than one fiber orientation, such as crossing or kissing fibers, or the branching of fiber pathways), the tensor model is inadequate as there is only one estimate of fiber orientation and reconstructed trajectory per voxel.

Probabilistic tractography is able to overcome these limitations by taking into account the local uncertainty in fiber orientation and repeating the streamline propagation process multiple times (Behrens et al., 2003b; Parker et al., 2003). Instead of a simple diffusion tensor, probabilistic algorithms repeatedly sample probability distribution functions (PDFs, generated based on uncertainty in eigenvector orientation) that describe the uncertainty of local fiber orientation distributions (e.g., see Tournier et al., 2008). During tract reconstruction, streamlines are propagated by randomly sampling from the distribution of fiber orientation estimates within each voxel and advancing the streamline in the direction of the interpolated modified principal eigenvector sampled. The streamline tracking process is repeated multiple times (usually in the order of ‘000s), with different samples drawn from the PDFs, generating a distribution of possible pathways. The number of times each voxel is reached by the advancing streamline is recorded, allowing for an estimation of connection probability from the seed region to any given voxel in the brain and a measure of the confidence which can be assigned to an identified route.

Since its initial development, a multitude of studies have been conducted aimed at furthering the diffusion tractography technique. These studies have focused on methodological refinements associated with the diffusion MRI sequence, the modeling of complex tissue fiber orientations, and the way in which the uncertainty in fiber orientation is derived and sampled (e.g., Assaf and Basser, 2005; Jones and Pierpaoli, 2005; Lazar and Alexander, 2005; Chung et al., 2006; Behrens et al., 2007; Dargi et al., 2007; Jian and Vemuri, 2007; Berman et al., 2008; Jones, 2008b; Wedeen et al., 2008; Haroon et al., 2009). Indeed, there is increasing evidence regarding the anatomical validity and reproducibility of the white matter pathways identified via the tractographic method and a number of pathways have been mapped which correlate strongly with those identified via tract tracing or post-mortem dissection techniques (Parker et al., 2002; Dyrby et al., 2007; Lawes et al., 2008). However, while substantial advancements continue to be made, important limitations still remain (for reviews, see Jones, 2008a; Mukherjee et al., 2008a,b; Johansen-Berg and Rushworth, 2009; Jones, 2010; Jbabdi and Johansen-Berg, 2011). The tractographic technique is currently incapable of identifying pathways at the axonal level due to limitations in the spatial resolution of the image acquisition (generally around 2 mm), and as such, can only be used to differentiate relatively large fiber bundles. The technique is also incapable of mapping extremely short-range intra-regional (horizontal) connections (although it should be noted that tract tracing methodologies also suffer from this limitation to some extent, due to the bleeding of the tracer at the injection site). In addition, tractography is indiscriminant as to the direction of connectivity and is incapable of differentiating between afferent or efferent pathways.

In tractography, the presence, absence and direction of neural fiber pathways is not visualized directly but must be indirectly inferred through water diffusion and computational analyses, and there are multiple sources of error which may affect the validity of the fiber pathways identified. Sources of error may arise during the acquisition of the diffusion MRI data due to noise, distortion artifacts and participant movement. In addition, modeling errors during tract reconstruction may also occur, resulting from factors such as partial volume effects and regions of complex fiber orientation, the branching of fiber pathways and the length and shape of the paths tracked. As such, there is a level of uncertainty in all tractographic data, with a degree of both false positives (Type I errors) and false negatives (Type II errors) inherent in any set of results. It has been argued, however, that the connectivity-based parcellation technique demonstrates a degree of robustness to tract modeling errors. Researchers have suggested that the connectivity patterns which serve as the input for cortical parcellation do not need to be a reflection of the complete underlying structural connectivity of the regions under examination, instead, what is of crucial importance is their sensitivity to any connectivity differences between regions (Jbabdi and Johansen-Berg, 2011; Knosche and Tittgemeyer, 2011).

### Connectivity-based parcellation methods

Cortical parcellation via white matter connectivity is based on the premise that, as connectional anatomy determines functional organization, regions with differing functional roles should demonstrate dissociable patterns of connectivity. As such, it should be possible to segregate functionally-distinct cortical regions based on their unique complement of corticocortical connections, parcellating the cortex by identifying boundaries where neural connectivity patterns change (Johansen-Berg et al., 2004; Behrens and Johansen-Berg, 2005; Anwander et al., 2007; Knosche and Tittgemeyer, 2011). Several different tractographic parcellation approaches have been developed but all share the same core processing stages. Connectivity profiles are first established for each subunit within the cortical region to be parcellated. These connectivity profiles (sometimes referred to as “connectional fingerprints”) are subsequently compared via various measures of similarity, and clustered into regions such that within-cluster subunits share highly similar patterns of connections, whilst between-cluster connectivity profiles are markedly distinct.

#### Connectivity profile determination

The first step in tractographic parcellation is the determination of a connectivity profile for each subunit within the region under examination. To establish a connectivity profile, tractography is initiated from each subunit “seed” within the cortical area being parcellated and the target brain regions connected to each seed are identified. Studies have varied in relation to the nature and number of the target brain regions used to establish these connectivity profiles. The two main approaches vary in relation to whether the profiles encompass information regarding connectivity from the seed to the whole brain (Johansen-Berg et al., 2004; Anwander et al., 2007; Tomassini et al., 2007; Mars et al., 2011, 2012) or a subset of key predetermined neuroanatomical regions (Behrens et al., 2003a; Johansen-Berg et al., 2005; Leh et al., 2007; Beckmann et al., 2009; Broser et al., 2011). The latter method benefits from its ability to infer functional roles to the delineated subregions and assess explicit structure-function hypotheses but may fail to discriminate more fine-grained functional subdivisions that may exist within the regions identified. In contrast, techniques which utilize whole-brain connectivity patterns have the potential to discern all anatomical/functional subdivisions within a cortical region and do not require any *a priori* hypotheses regarding the region's anatomical or functional organization.

Studies which have examined whole-brain connectivity profiles have traditionally defined the cortical targets as individual voxels (usually in the order of 2–5 mm^3^ in size; see e.g., Johansen-Berg et al., 2004; Klein et al., 2007), resulting in a substantial number of target regions contributing to the connectivity profile analyses. In contrast, studies which have utilized a set number of predefined regions have used larger but substantially fewer cortical targets (with as few as two target regions in some studies, e.g., Leh et al., 2007). The effect of varying the size/number of target regions on the resultant parcellations is currently unclear. In a study assessing the reproducibility of tractographic-based thalamic segmentations, Traynor et al. (2010) examined the impact of target region number on the parcellation outcomes, comparing connectivity profiles in which the cortical surface was divided into either 6 or 31 cortical targets. When only six large cortical targets were used, thalamic parcellations were produced which were comparable to those obtained by studies which utilized voxel-sized targets (e.g., Jbabdi et al., 2009). A similar parcellation was also obtained when the number of target regions was increased to 31, although some further subdivisions were observed. Importantly, when reproducibility of the tractographic segmentations were examined by analyzing 16 datasets from the same subject, the parcellations based on six target regions were found to be more consistent that those obtained using a larger number of target regions. This finding led Traynor et al. to conclude that when conducting parcellation studies involving multiple subject comparisons, a lower number of target regions may help to reduce inter-subject variability. The high inter-subject variability and lack of correspondence between functional subregions across individuals, has been viewed as a major limitation of the voxel-wise parcellation approach, resulting in a reduced interpretability of the results and an increased difficulty in determining the validity of the parcellations obtained (Jbabdi et al., 2009). This reduced reproducibility across individuals also has important implications in the use of tractographic parcellations in the assessment of potential neuroanatomical changes in different disease states. As such, while voxel-wise parcellation methods benefit from the ability to identify subtle differences between connection pathways, termination points between regions, and to produce more fine-grained subdivisions (which may potentially be a truer reflection of the brain's underlying anatomical and functional architecture), this may be at the cost of decreased reproducibility. As such, when determining the nature of the cortical target regions used during connectivity profile analyses, it is important to consider the purpose and goal of the parcellation conducted.

#### Clustering and determination of parcellation solutions

Once the connectivity profiles are established for each subunit within the area to be parcellated, similarity metrics are calculated and used to cluster the subunits so that neighboring regions with similar connectivity patterns are combined. To date, tractographic parcellation studies have utilized two main clustering methods, namely, spectral reordering (e.g., Johansen-Berg et al., 2004; Devlin et al., 2006; Solano-Castiella et al., 2010), and *k*-means cluster analysis (e.g., Anwander et al., 2007; Klein et al., 2007; Tomassini et al., 2007; Nanetti et al., 2009; Gorbach et al., 2011; Mars et al., 2012).

One of the first studies to utilize white matter tractography for connectivity-based *in vivo* parcellation was conducted by Johansen-Berg et al. (2004), who used spectral reordering to parcellate the human medial frontal cortex into supplementary and pre-supplementary motor area (SMA/pre-SMA) subdivisions. In the spectral reordering technique, connectivity profiles are transformed into a symmetric cross-correlation matrix, which represents correlations in connectivity patterns between each subunit in the cortical region being parcellated. A spectral reordering algorithm (Barnard et al., 1995) is then applied, which imposes a structural organization to the cross-correlation matrix such that larger values (i.e., high correlations) are forced towards the diagonal. Consequently, subunits with similar connectivity are positioned together within the matrix, forming visually distinguishable clusters. In the final stage of parcellation, the clusters are segregated by visual inspection into connectionally-distinct cortical subregions. A major advantage of the spectral reordering technique is that it does not require any *a priori* assumptions regarding the number of clusters present within the region being parcellated (as with *k*-means, see below). As such, it is very useful for exploratory analyses when the underlying anatomical structure of the region being parcellated is largely unknown. However, cluster member allocation and final parcellation is performed manually by visual analysis and is not based on any statistical criterion, introducing a lack of objectivity in the determination of break point locations within the reordered matrices. The impact of this on inter-rater reliability and inter-subject variability has yet to be empirically assessed. In addition, the clusters within the cross-correlation matrix may not always show clear divisions and, as such, the visual segregation may not always be a clear and straightforward process.

An alternative approach which does enable the objective identification of connectivity-based structural boundaries is *k*-means cluster analysis (Anwander et al., 2007; Klein et al., 2007). *K*-means clustering is an iterative algorithm which divides the (voxel) subunits within the area being parcellated into *k* non-overlapping clusters. Within *k*-means, each subunit is characterized as a point whose coordinates represent the correlation of its connectivity profile with those of every other subunit. Parcellation begins with the random assignment of *k* initial cluster centers (centroids), which may be performed by the selection of random subunits from within the initial dataset (Hartigan and Wong, 1979) or by splitting the data into subsets of relatively equal size and determining their respective baricenters (Lloyd, 1982). Each subunit within the region is then assigned to the nearest centroid, with grouping performed by minimizing the sum of squares of distances between each subunit and the corresponding cluster centroid. The centroids of the newly formed clusters are recalculated and the subunits are subsequently grouped again to the closest cluster centroid, with this process repeated until centroid positions change less that a predetermined threshold. Although more objective than cluster determination by visual inspection, there are two important disadvantages to the *k*-means parcellation approach. Firstly, the algorithm requires the *a priori* specification of the number of clusters to be parcellated. This selection is often based on prior anatomical knowledge or the cluster number which yields the most topologically consistent results across subjects (e.g., Beckmann et al., 2009; Mars et al., 2011, 2012). Secondly, the algorithm is sensitive to the assignment of initial cluster centers and the repeated application of *k*-means clustering to the same dataset can lead to variable parcellation solutions (Nanetti et al., 2009). This was explored directly by Nanetti et al. (2009), who observed that the repeated use of *k*-means clustering produced different cortical parcellations depending on the initial placement of the start point, and found that to estimate the distribution of possible solutions accurately, approximately 250 *k*-means repetitions were required.

One final clustering technique, which provides both objective cluster assignment and does not require any *a priori* assumptions regarding the number of clusters, is hierarchical cluster analysis (Johnson, 1967). Within this approach, each subunit is initially assigned to a cluster such that each individual subunit constitutes a single cluster. A hierarchy is then created by progressively merging the most similar clusters (i.e., those with the most similar connectivity profiles), producing successively fewer but larger clusters until all subunits are grouped into a single cluster. This hierarchy has been argued to represent the organizational structure from the level of individual units to the cortical surface comprising the region being parcellated (Eickhoff et al., 2011). A major advantage of the hierarchical approach is that it allows parcellation at various levels of the hierarchy, producing *k* clusters by cutting the *k*-1 longest linkages. Conversely, there may be no clear delineation of the number of segregated regions within the cortical area being parcellated and the final parcellation solution may be somewhat arbitrarily selected. To date, the hierarchical approach has not been commonly implemented in tractographic parcellations, however, it has been used to cluster connectivity profiles within the primate brain identified via tracer data (Kotter et al., 2001a; Passingham et al., 2002). The technique has also been used to delineate structural/functional borders within the human brain via meta-analytic parcellation methods, for which it has been found to produce parcellation solutions comparable to those obtained using spectral reordering methods (Eickhoff et al., 2011).

The discussion above has presented just a few of the different methods which may be utilized to delineate structural boundaries within the cortex based on patterns of corticocortical connectivity. A number of alternative methodological approaches have recently been developed including Dirichlet process mixture models (Jbabdi et al., 2009), fuzzy *k*-means clustering (Mars et al., 2012), hierarchical information-based clustering (Gorbach et al., 2011), independent component analysis (O'Muircheartaigh et al., 2011), force-direct graph layout (Crippa et al., 2011), and spatially-informed dimension reduction (Roca et al., 2009). Although varying in the specific statistical algorithms used to determine cluster formation, all attempt to form coherent cortical subregions by minimizing the variability in connectivity patterns within clusters while maximizing connective variability between them. Given its potential utility in the study of the structural and functional organization of the brain *in vivo*, a substantial amount of research has recently been conducted to explore the efficacy and validity of the tractographic parcellation approach. This research will be explored in the following section.

## Validation of tractographic connectivity-based parcellations

White matter connectivity profiles derived from tractography have been used to parcellate a rapidly increasing number of cortical structures including the medial frontal cortex (SMA/pre-SMA; Johansen-Berg et al., 2004; Lehericy et al., 2004; Klein et al., 2007; Jbabdi et al., 2009; Nanetti et al., 2009; Crippa et al., 2011), inferior frontal cortex (Anwander et al., 2007; Klein et al., 2007; Gorbach et al., 2011), precentral gyrus (Tomassini et al., 2007; Schubotz et al., 2010; Gorbach et al., 2011), postcentral gyrus (Roca et al., 2010), inferior parietal cortex (Mars et al., 2011, 2012), temporoparietal junction (Mars et al., 2012), insula (Nanetti et al., 2009; Jakab et al., 2012), and cingulate cortex (Beckmann et al., 2009), as well as a number of subcortical structures including the thalamus (Johansen-Berg et al., 2005; Devlin et al., 2006; Traynor et al., 2010; Broser et al., 2011; O'Muircheartaigh et al., 2011; Elias et al., 2012), subthalamic nucleus (Lambert et al., 2012), basal ganglia (Lehericy et al., 2004; Leh et al., 2007; Draganski et al., 2008), amygdala (Solano-Castiella et al., 2010; Bach et al., 2011) and the substantia nigra (Menke et al., 2010). These studies provide increasing evidence that white matter tractography is indeed capable of identifying distinct connectivity profiles between cortical regions, and that these differences may be used to differentiate structurally (and by inference, functionally) distinct subregions. Nevertheless, the parcellation schemes derived are the product of an indirect measure of the underlying neural structure which is not visualized directly, as with more traditional microstructural methods. As such, it of great importance to substantiate the identified parcellations in relation to their consistency and variability, and determine the degree to which these connective boundaries correspond to the functional borders they are hypothesized to reflect, as well as their relationship with more traditionally established (micro)structural architectures.

Tractographic parcellations of the medial frontal cortex have consistently identified two principal subregions, a posterior subdivision which corresponds well to the SMA and an anterior subdivision which corresponds to the pre-SMA (e.g., Johansen-Berg et al., 2004). In order to assess the within- and between-subject consistency of parcellation solutions produced using the *k*-means approach, Nanetti et al. (2009) repeated the tractographic parcellation of the SMA/pre-SMA 1000 times. The number of clusters for all iterations was predefined as *k* = 2. A substantial degree of variability was observed in the location of regional borders both within and across individuals, with only one out of the ten participants demonstrating identical solutions for all 1000 parcellations. For the remaining nine participants, any single solution accounted for at best 75% of all possible solutions, with as many as six different solutions found for a given individual. It appears that for *k*-means at least, a degree of variability is inherent in the approach and so a firm conclusion regarding the precise location of inter-regional borders cannot be inferred by a single parcellation. Instead, as noted above, multiple repetitions may be required to establish a probability distribution of solutions from which the most likely boundary locations may be established.

For one participant in the Nanetti et al. (2009) study, a parcellation solution was observed which was anatomically improbably, with one cluster constituting non-contiguous anterior-most and posterior-most cortical areas, and the second cluster taking a position between these two regions. It was suggested that for a number of individuals, the medial frontal area may actually constitute three rather than two distinct regions. Indeed, for one participant who demonstrated four variable solutions at *k* = 2, when the number of clusters was increased to *k* = 3, only a single solution was observed. The existence of a third region between the two principal SMA/pre-SMA areas has also been identified for a subset of participants in other tractographic parcellation studies (Johansen-Berg et al., 2004; Crippa et al., 2011). The consistent observation of three subregions across participants and studies suggests that this parcellation scheme may have anatomical reality. It has been suggested that the region may reflect a “transitional” zone of hybrid connectivity between the two areas (Crippa et al., 2011). This finding raises two important issues in relation to connectivity-based parcellations particularly, as well as cortical parcellation more generally. Firstly, it reiterates the difficulty in the *a priori* specification of cluster numbers, either by prior anatomical knowledge or inter-subject consistency, highlighting the importance of techniques which do not require such assumptions (such as spectral reordering or Dirichlet process mixture models; Johansen-Berg et al., 2004; Jbabdi et al., 2009). Secondly, the majority of parcellation techniques force a solution that is unique, complete, and which delineates hard borders between regions (Gorbach et al., 2011; Knosche and Tittgemeyer, 2011). However, there is strong evidence to suggest that this may not reflect anatomical reality. For example, anatomical studies of the insula have observed an anteroventral-posterodorsal gradient across both the region's underlying cytoarchitecture (Mesulam and Mufson, 1982) and neural connectivity (Cerliani et al., 2012; Cloutman et al., 2012) with the presence of an intermediary transition zone between its ventral-anterior agranular and dorsal-posterior granular regional extremities. Such transition areas may contribute to the variability in border location and number of subregions observed across individuals. As a consequence, researchers have begun to develop tractographic parcellation techniques which enable soft partitioning (Gorbach et al., 2011).

Despite these issues, there is increasing evidence to indicate that the tractographic parcellation technique can indeed identify structural subregions and border locations which correspond strongly with a region's proposed functional organization. In an attempt to assess the functional validity of the tractographically-defined SMA/pre-SMA boundaries identified, Johansen-Berg et al. (2004) conducted an fMRI study in which participants were required to perform either blocks of finger tapping (preferentially activating the SMA; Rao et al., 1993; Eickhoff et al., 2011) or a serial subtraction task (preferentially activating the pre-SMA; Johansen-Berg and Matthews, 2002; Arthurs et al., 2004). A strong anatomical correspondence between the tractographic subregions and observed fMRI activations was found, with the connectively-defined SMA/pre-SMA cluster centers co-localizing closely with those of the corresponding SMA/pre-SMA functional activation clusters (within 2–3 mm; see also Klein et al., 2007). Further support for this structure-function relationship comes from a recent functional parcellation study which used meta-analytic connectivity mapping (which defines subregions on the basis of patterns of functional co-activation), to segregate the medial frontal cortex into SMA/pre-SMA regions which aligned closely with those previously defined tractographically (Eickhoff et al., 2011). A similar correspondence between tractographic subregions and functional activations have also been observed for other regions of the brain, such as the precentral gyrus, for which anatomical parcellations have been found to predict the distribution of functional activations (Tomassini et al., 2007; Schubotz et al., 2010). Indeed, the relationship between tractographic and function subdivisions has been found even within regions demonstrating a high degree of anatomical and functional complexity. For example, Beckmann et al. (2009) identified nine tractographic subregions within the cingulate cortex which were consistently related to regions of functional specialization as revealed by meta-analysis.

An important advantage of the tractographic parcellation approach is that it not only provides information regarding the location and boundaries of subregions within a given area, but also enables functional roles to be ascribed to the identified subregions based on their patterns of corticocortical connections. The examination of cortical targets provides additional convergent evidence regarding the functional validity of the tractographic subregions identified. For example, tractographic parcellation of the cingulate cortex revealed a subregion within the anterior cingulate gyrus dorsal to the corpus callosum (cluster 7, Beckmann et al., 2009). A meta-analysis revealed this region to be associated with emotion and social behavior, which was consistent with its observed connectivity with the hypothalamus, amygdala, ventral striatum, and orbitofrontal cortex. Furthermore, investigation of the corticocortical connectivity of the subregions delineated also enables functional roles to be (tentatively) inferred in regions where function remains poorly understood (see the discussion of the tractographic parcellation of the inferior parietal cortex below).

## Tractographic versus cytoarchitectonic cortical parcellations

Both cellular microstructure and neural connectivity are heavily implicated in determining the functional specialization of a region. Indeed, cytoarchitecture has dominated parcellation schemes of the cerebral cortex becoming the “gold standard” for the delineation and segregation of anatomical subregions (see Amunts et al., 2007, for a review). A key advantage of cytoarchitectonic parcellations is that structural boundaries are visualized directly and are not based on an indirect delineation of the underlying neural structure. Classic cytoarchitectonic approaches, such as that utilized by Brodmann (1909), are well known to have suffered from inaccuracies due to a lack of observer independency, reproducibility and objectivity in the segregation of regions (see Zilles and Amunts, 2010, and Geyer et al., 2011, for a review). Modern cytoarchitectonic techniques have made it possible not only to parcellate with much greater spatial resolution but also to compare the results across specimens, thereby generating probabilistic cytoarchitectonic maps (Schleicher et al., 1999, 2005; Eickhoff et al., 2005a; Amunts et al., 2007). Using the volume density of cell bodies (gray level index, GLI) to define boundaries and multivariate analyses to identify changes in lamination patterns, automated observer-independent cytoarchitectonic parcellations have been undertaken on many areas of the brain (e.g., Amunts et al., 1999, 2000; Geyer et al., 1999, 2000; Morosan et al., 2001; Caspers et al., 2006, 2008). Consequently, to assess the accuracy and validity of tractographic parcellations, studies have sought to compare the results from connectivity-based parcellations with those identified cytoarchitectonically.

A substantial proportion of tractographic parcellation studies have attempted to provide anatomical validation of the derived parcellation schemes by comparison with the expected regional distribution and areal volume as predicted by modern probabilistic cytoarchitectonic maps (e.g., Johansen-Berg et al., 2005; Devlin et al., 2006; Anwander et al., 2007; Klein et al., 2007; Broser et al., 2011; Gorbach et al., 2011; Mars et al., 2011, 2012; Jakab et al., 2012). These studies have, in general, found a relatively strong correspondence between the parcellation solutions produced by the two methodologies. For example, in their tractographic parcellation of the inferior frontal cortex (Broca's area, BAs 44/45), Klein et al. (2007) observed that both the *k*-means clustering and spectral reordering tractographic approaches were able to recover 90–95% of the “gold-standard” (cytoarchitectonic) segmentation. Similar correspondence was also observed by Anwander et al. (2007), who found a strong coincidence between the center of gravity coordinates for the tractographic and cytoarchitectonic BA 44/45 clusters, although the connectively-defined regions were found to have a reduced *z* extent. However, while the two tractographic regions demonstrated strong regional overlap with the cytoarchitectural regions, being completely included within the areal boundaries of the respective probabilistic maps, they were observed to be volumetrically smaller than their architectonic counterparts.

The strong correspondence observed between cortical subregions defined connectively and those defined cytoarchitectonically has been used to provide external validation of the tractographic parcellation approach. The focus on the use of cytoarchitecture as the gold standard has been based on the underlying assumption that a strong degree of correspondence exists between the two neuroanatomical features and, consequently, the anatomical segmentations they produce. Primate studies have observed that functionally and cytoarchitectonically distinct brain regions appear to be associated with distinct corticocortical connection patterns, suggesting a strong relationship between brain connectivity and cellular microstructure (Jones and Burton, 1976; Cavada and Goldman-Rakic, 1989; Kotter et al., 2001a; Passingham et al., 2002; Blatt et al., 2003; Rozzi et al., 2006; Petrides and Pandya, 2009; Gerbella et al., 2010). Indeed, some researchers have gone as far as to propose that corticocortical connectivity patterns may be used as an indirect *in vivo* surrogate marker of cytoarchitectural divisions and boundaries (e.g., Klein et al., 2007). Although cytoarchitectonic- and connectivity-based parcellations have been found to demonstrate strong congruence for a number of the cortical areas examined to date, this is not always the case. For example, a recent tractographic parcellation study of the insula revealed that changes in connectivity profiles across the area did not respect either the underlying cytoarchitecture or gyral anatomy (Jakab et al., 2012). Differences in cytoarchitectural-connectional correspondence were also observed in a recent study by Mars et al. (2011, 2012), which tractographically parcellated the right inferior parietal cortex into five connectionally-distinct subregions. While these tractographic subregions appeared to bear a strong correspondence to those recently delineated via cytoarchitecture, notable differences were observed. Specifically, while the cytoarchitectonic parcellation delineated seven distinct subregions (see Caspers et al., 2006, 2008), only five tractographic subregions were identified, with the right supramarginal gyrus partitioned into only three (as opposed to five) connectivity-based subdivisions. Within this tractographic parcellation, not all cytoarchitectonic regions demonstrated unique neural connectivity, and several cytoarchitectonic areas were clustered together to form larger functional subdivisions based on similarities in their connectivity profiles and associated fiber tracts. This is consistent with evidence from tract tracing studies in primates, and Passingham et al. (2002) noted that while no two cytoarchitectonic regions exhibited identical patterns of neural connectivity, it was possible to detect clusters of cytoarchitectonic regions with highly similar connectivity profiles, which these researchers referred to as “connectional families.” It seem likely that the different cytoarchitectonic regions comprising these connectional families are components of similar functional brain networks, playing-related and/or shared roles in an overlapping set of cognitive functions. As such, the relationship between cytoarchitectonic and tractographic parcellations may not necessarily be one-to-one, and connectivity profiles may not always directly overlap and delineate all cytoarchitectonic regions. Furthermore, there is evidence from primate tracer studies to indicate that the converse may also be true, and patterns of neural connectivity may be used to parcellate architectonically homogenous subregions into more fine-grained subdivisions (Felleman and Van Essen, 1991; Hilgetag and Grant, 2000).

From the above discussion it is apparent that the relationship between cytoarchitecture and connective architecture is complex, and the degree of correspondence and relative functional importance of cytoarchitectonic versus tractographic cortical parcellations still remains unclear. Cytoarchitecture and connectivity are complementary neural architectures which both contribute strongly to the functioning of a given brain region, and the focus on one without reference to the other is likely to leave our understanding of the structural-functional organization of the brain incomplete. As such, a key focus for future research is in the integration of information provided by the two methodologies, and the multimodal characterization of cortical areas.

## Alternative parcellation methodologies

While, the current review has focused on the use of neural connectivity (as identified via diffusion tractography), and cytoarchitecture in the delineation of structural and functional subdivisions within the brain, a number of alternative techniques exist. Indeed, cellular lamination patterns and white matter connectivity profiles are but two of the brain's architectural elements which may be used to demarcate regions of specialization. Myelination patterns, neurotransmitter receptor distributions, and (immuno)histochemical properties may all provide additional important information regarding brain organization and function, and have been utilized to provide myelo-, recepto-, and chemoarchitectonic cortical parcellations (e.g., Gerbella et al., 2007; Belmalih et al., 2009; Gallay et al., 2012; for reviews see Zilles et al., 2002b; Geyer et al., 2011). Furthermore, researchers have suggested that in addition to structural connectivity, regions of functional coherence may also be identified via their functional connectivity (e.g., Cohen et al., 2008; Kim et al., 2010; Mars et al., 2011, 2012). These alternative techniques will briefly be discussed.

### Alternative neuroarchitectural parcellation techniques

The anatomical microstructure of the brain is highly complex, and can be viewed at multiple, increasingly fine-grained architectural scales. At the larger scales are cell types and their patterns of distribution across laminae (cytoarchitecture), as well as the degree and disposition of intracortical axonal myelination (myeloarchitecture). While the majority of myelin resides within the axonal fiber bundles composing the brain's white matter, a substantial amount can be found within the cortical grey matter, associated with the small local fiber bundles which run parallel and perpendicular to the cortical sheet (Annese et al., 2004; Bock et al., 2011). Importantly, the density and arrangement of these intracortical myelinated fibers have been found to show striking variation across the cortical mantle, and histological myelin staining has been able to reveal clear myeloarchitectonic differences between regions of the cortex (Zilles and Palomero-Gallagher, 2001; Geyer et al., 2011). At a more fine-grained scale, differences can also be observed at the synaptic level in relation to the type and distribution of neurotransmitter receptor binding sites (receptoarchitecture). *In vitro* autoradiography techniques have revealed that the balance of different receptor (sub)types can vary both in relation to their relative concentrations between different cortical regions, as well as their distribution across different cortical layers within a given region (Zilles et al., 2004; Scheperjans et al., 2005b; Eickhoff et al., 2007). As with cytoarchitecture, intracortical myelination and neurotransmitter specificity are heavily implicated in the functioning of a given neuronal population. For example, glutamate is one of the brain's predominant excitatory neurotransmitters, while in contrast, GABA has an inhibitory function, inhibiting action potential firing (Purves et al., 2004; Jacobson and Marcus, 2008). As such, differences in myelo- and recepto-architecture between cortical regions have been postulated to mirror functional differences between areas (Scheperjans et al., 2005b; Eickhoff et al., 2007).

Although much less prevalent that cytoarchitectural parcellations, numerous studies have attempted to segregate the cortex into regions of functional sub-specialization via myelo- and receptor-architectonics, successfully parcellating areas within the parietal cortex (Scheperjans et al., 2005a, 2008), temporal cortex (Rivier and Clarke, 1997; Wallace et al., 2002; Padberg et al., 2003; Morosan et al., 2005; Ding et al., 2009), prefrontal cortex (Carmichael and Price, 1994; Ongur et al., 2003; Gerbella et al., 2007; Belmalih et al., 2009; Cruz-Rizzolo et al., 2011), visual cortex (Zilles et al., 2004), motor and somatosensory cortices (Zilles et al., 1995; Geyer et al., 1997), insula (Rivier and Clarke, 1997; Gallay et al., 2012), and subcortical structures including the amygdala (Sims and Williams, 1990). These studies have consistently observed a strong correspondence between the cyto-, myelo-, and receptoarchitectonically defined areal borders, providing strong indication that these anatomical properties are indeed identifying structurally and functionally coherent subdivisions of the brain (Sims and Williams, 1990; Zilles et al., 1995, 2002a). For example, Geyer et al. (1997) observed that changes in regional and laminar neurotransmitter receptor distribution patterns within the human primary somatosensory cortex were able to define borders between areas 4 and 3a, areas 3b and 1, and areas 1 and 2, and noted that the location of these areal boundaries mapped precisely onto those defined via cytoarchitecture. In addition, correlations with connectivity-based parcellations have also been observed. For example, Kotter et al. (2001b) directly compared receptoarchitectonic parcellations to those delineated via connectivity (identified via tract tracing techniques) in the macaque motor and visual cortices. While some partial discrepancies were observed, notably within the visual cortex, a strong correspondence was found between the two parcellation methodologies. However, while a strong association between myelo- and cyto-architectural regional divisions has consistently been observed, suggesting that myeloarchitecture may be able to define cytoarchitectonic boundaries, receptoarchitectonic parcellations have differentiated additional regional subdivisions not identified via the other methodologies (Geyer et al., 1997; Zilles et al., 2004). Thus, receptoarchitecture appears to be a powerful methodology for delineating increasingly fine-grained maps of functional specialization within the cortex.

However, receptoarchitecture is only observable post-mortem, limiting its utility in the exploration of functional specialization within the human brain. In contrast, recent studies have demonstrated that it is possible to delineate the brain's myeloarchitecture *in vivo* using high field (i.e., 7T) MRI (Walters et al., 2003; Bridge et al., 2005; Eickhoff et al., 2005b; Bock et al., 2011; Glasser and Van Essen, 2011; Cohen-Adad et al., 2012). Sharp transitions in myelin content across the cortical surface, representative of regional borders, have been identified via MRI in a number of regions including the primary motor (BA 4), somatosensory (BA 3), and visual cortices (BA 17), as well as the auditory association cortex (BA 42), and Broca's area (BA 44/45; Bridge et al., 2005; Cohen-Adad et al., 2012). These MRI-based cortical myelination patterns have been found to correspond closely to the myelin distributions identified histologically within the same brains (Osechinskiy and Kruggel, 2009). In addition, the myeloarchitectonic maps delineated have been found to demonstrate substantial overlap with those defined via probabilistic cytoarchitecture (Glasser and Van Essen, 2011). Importantly, there is also evidence that these MRI-based myeloarchitectonic subdivisions correspond to functional subdivisions as identified by fMRI. Bridge et al. (2005) compared structurally- and functionally-defined borders within the human visual cortex, and found that the delineation of the V1/V2 functional boundary identified via fMRI showed a close correspondence to the myeloarchitectonic anatomical boundary identified via a T1-weighted MRI scan.

The visualization and delineation of myeloarchitecture via high-resolution MRI is only beginning to be explored, and a substantial amount of research is required before it can become a reliable method for the identification of structural and functional subdivisions within the brain. Still, it shows great potential as a powerful tool for cortical parcellation, and in combination with fMRI and diffusion tractography, may enable the exploration of the relationship between local microstructure, long-range connective architecture, and cognitive functioning *in vivo*.

### Functional connectivity parcellation techniques

Structural white matter connections are but one form of long-range “connectivity” which may be delineated within the brain *in vivo*, and researchers have begun to identify regions of functional specialization within the cortex via patterns of functional connectivity (e.g., Cohen et al., 2008; Karkar et al., 2009; Kim et al., 2010; Zhang et al., 2010; Craddock et al., 2012; Yeo et al., 2011). Spontaneous low frequency fluctuations are evident within the fMRI signal when the brain is at “rest” (i.e., non-task dependent). Importantly, these intrinsic activity fluctuations are not random and show temporal correlations between functionally connected regions, that is, functional connectivity (for reviews see Fingelkurts et al., 2005; Buckner and Vincent, 2007; Fox and Raichle, 2007; Buckner, 2010; Cole et al., 2010; Margulies et al., 2010; van den Heuvel and Hulshoff Pol, 2010; Deco and Corbetta, 2011; Raichle, 2011; Kelly et al., 2012a). It has been postulated that these patterns of intrinsic functional connectivity are a consequence of the repeated evoked coactivation of regions during the execution of functional tasks, and reveal a structured spatio-temporal organization which reflects the underlying functional organization of the brain (Deco and Corbetta, 2011; Kelly et al., 2012b).

By identifying brain regions whose intrinsic activation fluctuations demonstrate strong correlations within the resting-state fMRI time-series, it is possible to delineate widely distributed, functionally coherent brain networks (Friston et al., 1993; Biswal et al., 1995; McKeown et al., 1998; Beckmann et al., 2005; Fox et al., 2005). Importantly, these functional connectivity patterns have been found to reveal sharp transitions in correlation patterns between neighboring regions, demarcating regional boundaries (Cohen et al., 2008; Yeo et al., 2011). Thus, the functional connectivity profiles of areas within a given brain region can be delineated and used to segregate the cortex into functionally specialized subdivisions using similar methods to those used for tractographic structural connectivity parcellations (e.g., *k*-means clustering). Using such methods, studies have successfully parcellated an increasing number of brain regions via their patterns of functional connectivity, including the frontal (Kelly et al., 2010; Kim et al., 2010; Kahnt et al., 2012), parietal (Margulies et al., 2009; Nelson et al., 2010; Barnes et al., 2011), temporal (Mars et al., 2012), and cingulate cortices (Margulies et al., 2007; Yu et al., 2011), as well as the insula (Cauda et al., 2011; Chang et al., 2012; Kelly et al., 2012b), cerebellum (Buckner et al., 2011), and subcortical structures including the thalamus (Zhang et al., 2008, 2010), striatum (Di Martino et al., 2008), and amygdala (Roy et al., 2009).

However, a parcellation scheme is only as accurate as the connectivity profiles upon which it is based, and there are a number of important caveats regarding the use of resting-state functional connectivity in the delineation of cortical subregions. Firstly, the underlying neuroanatomical basis driving intrinsic fluctuations in brain activity remain poorly understood, and there is some evidence to suggest that at least some may have a non-neuronal origin (Wise et al., 2004; Birn et al., 2006, 2008a,b; Chang et al., 2009; Van Buuren et al., 2009). Importantly, it has been observed that physiological processes unrelated to neuronal function, such as respiratory and cardiac cycles, may introduce artificial correlations in the fMRI signal fluctuations between distributed brain regions. For example, Wise et al. (2004) demonstrated that regions within the occipital, parietal, and temporal lobes exhibited oscillations within the fMRI time-series which covaried significantly with fluctuations in levels of arterial carbon dioxide. A number of strategies have been developed to address the issue of these non-neuronal contributions, such as utilizing fast sampling rates (Chuang and Chen, 2001), and post-processing data analysis methods including independent component analysis (Kiviniemi et al., 2003; Beckmann et al., 2005), and global signal regression (Zarahn et al., 1997; Macey et al., 2004). However, the optimal strategy to deal with this physiological noise has yet to be identified, and it has been noted that the analytic approach used can have a significant impact on the spatial characteristics of the functional connectivity networks delineated (Murphy et al., 2009; Cole et al., 2010). A second important issue in relation to the validity of functional connectivity-based cortical parcellations is in relation to the reliability of the resting-state functional connectivity networks delineated. A strong consistency has been found between the functional connectivity networks identified across individuals and datasets, and a moderate to high test–retest reliability has been observed (Buckner et al., 2009; Shehzad et al., 2009; Biswal et al., 2010; Van Dijk et al., 2010; Zuo et al., 2010a,b). However, some questions have been raised. For example, Honey et al. (2009) examined the correlation strengths between region pairs across multiple data sets collected on the same individuals, and observed only low to moderate within-subject reliability (*r* = 0.38–0.69). In addition, studies have identified that some network connections demonstrate greater reproducibility than others, with connectivity between regions composing the “default network” produced more reliably than those associated with the “task positive” network (Shehzad et al., 2009; Meindl et al., 2010).

While a number of studies have begun to utilize connectivity information from both diffusion tractography and resting-state functional connectivity, few have attempted to directly compare the cortical parcellation schemes derived via the two methodologies. One study which did undertake this endeavor was conducted by Zhang et al. (2010), who explored the structural and functional connectivity of the thalamus. Subregions within the thalamus were readily observable based on their differential patterns of connectivity with five different cortical regions, with similar patterns and strong anatomical overlap found between the tractographic and functional profiles. In addition, a good concordance was observed between these connectivity-based parcellations and those defined histologically (see also Zhang et al., 2008). However, differences between the structural and functional mappings were identified, and functional connections were observed which did not correspond to those delineated via tractographic methods. Indeed, the relationship between structural and functional connectivity is complex, and their mapping is by no means one-to-one (Koch et al., 2002; Rykhlevskaia et al., 2008; Skudlarski et al., 2008; Uddin et al., 2008; Damoiseaux and Greicius, 2009; Greicius et al., 2009; Honey et al., 2009, 2010; Van Dijk et al., 2010; Tyszka et al., 2011). Studies have identified strong relationships between functional and structural connectivity, with the presence of a direct anatomical connection between two regions associated with the presence of strong functional connections (Skudlarski et al., 2008; Honey et al., 2009, 2010). However, functional connectivity does not necessarily infer structural connectivity, and studies have observed functional connections between regions where no structural connectivity exists (Uddin et al., 2008; Zhang et al., 2008; Tyszka et al., 2011). For example, inter-hemispheric functional connections have been identified in individuals with agenesis of the corpus callosum (Tyszka et al., 2011), or after undergoing commissurotomy (Uddin et al., 2008). Conversely, brain regions which have been shown to be strongly anatomically connected may demonstrate only weak functional connectivity (Skudlarski et al., 2008). Thus, it seems apparent that the brain's structural and functional connectivity, while complementary, is not identical. How the two connectivity methodologies relate to each other, and their relative importance in the delineation of regions of functional specialization within the cortex is an important issue which is only beginning to be explored.

As noted above, functional connectivity represents regions with correlated patterns of activation fluctuation, and while structural connectivity strongly constrains functional connectivity, functional coupling between two brain regions may occur via direct anatomical connections, or through indirect structural connections via shared brain regions (Damoiseaux and Greicius, 2009). As such, the interpretation of the underlying nature and meaning of a functional connection between two brain regions is less straightforward than for anatomical connections (Rubinov and Sporns, 2010; Sporns, 2011). Still, when the ultimate goal of cortical parcellation is in the delineation of regions of functional specialization, it could be proposed that functional connectivity profiles benefit from the fact that they are indeed “functional”. However, it has been noted that like diffusion tractography, resting-state functional connectivity is limited in its functional interpretation as it carries no task-related information (Eickhoff et al., 2011). Indeed, while a strong overlap between the brain's intrinsic functional networks and those activated during task performance have been identified, this has not been consistently observed, and weak coupling between intrinsic and extrinsic functional architectures have been noted in subcortical, sensory, and motor regions (Mennes et al., 2012). As such, a resting-state functional connection does not necessarily relate to a task-based functional connection. Consequently, researchers have developed new task-based functional connectivity approaches, such as meta-analytic connectivity modeling, which show initial promise in delineating functionally interpretable parcellations of the human cortex (Eickhoff et al., 2011; Cauda et al., 2012).

## Conclusion

“The theory of anatomical localization is, like physiological and clinical localization, still in a state of development. The directions to take to reach them are not immediately attainable. So many problems must remain unresolved, some can only be provisionally unravelled, and for yet others the way ahead can only be sketched out roughly.”

Brodmann (1909, p. 2)

Since these words were written by Brodmann, the landscape of neuroanatomical research, and the methodological techniques available to visualize and map the cerebral cortex have advanced exponentially. However, despite over 100 years of research, their sentiment still rings true and there is currently no consensus as to what constitutes a cortical region or what criteria and anatomical features should be used in their delineation (Belmalih et al., 2009).

Post-mortem histological identification of cytoarchitecture has been one of the most prominent methodologies for the delineation of structurally and functionally meaningful subdivisions of the brain, made famous by Brodmann himself. Brain function strongly depends upon both anatomical microstructure and connective architecture–cytoarchitecture determines a region's local processing capabilities whilst its connectivity governs the nature and flow of information to and from an area. The advent of modern diffusion imaging and white matter tractography has enabled the parcellation of the cortex into functionally specialized subregions via their unique complement of corticocortical connections *in vivo*. Recent studies have provided strong evidence in support of the efficacy of this connectional parcellation methodology, successfully parcellating areas including the inferior frontal cortex (Anwander et al., 2007), the supplementary and pre-supplementary motor areas (Johansen-Berg et al., 2004), the premotor cortex (Tomassini et al., 2007), and the thalamus (Johansen-Berg et al., 2005), emphasizing the relationship between variations in functional specialization and the underpinning connectional architecture. While important limitations still remain regarding both the tractographic parcellation technique and the delineation of the white matter pathways which serve as its input, substantial methodological advances continue to be made, improving its accuracy, validity and utility in the study of cortical functioning. The current research focus on the parcellation of the cerebral cortex via patterns of neural connectivity echoes Brodmann's original observations and his call for ‘fibrilloarchitectonics’, which modern diffusion tractography can now deliver, *in vivo*.

### Conflict of interest statement

The authors declare that the research was conducted in the absence of any commercial or financial relationships that could be construed as a potential conflict of interest.

## References

[B1] Amunts K. Malikovic A. Mohlberg H. Schormann T. Zilles K. (2000). Brodmann's areas 17 and 18 brought into stereotaxic space – Where and how variable? Neuroimage 11, 66–84 10.1006/nimg.1999.0516 10686118

[B2] Amunts K. Schleicher A. Burgel U. Mohlberg H. Uylings H. B. M. Zilles K. (1999). Broca's region revisited: cytoarchitecture and intersubject variability. J. Comp. Neurol. 412, 319–341 10.1002/(SICI)1096-9861(19990920)412:2<319::AID-CNE10>3.0.CO;2-7 10441759

[B3] Amunts K. Schleicher A. Zilles K. (2007). Cytoarchitecture of the cerebral cortex – More than localization. Neuroimage 37, 1061–1065 10.1016/j.neuroimage.2007.02.037 17870622

[B4] Angelucci A. Clasca F. Bricolo E. Cramer K. S. Sur M. (1997). Experimentally induced retinal projections to the ferret auditory thalamus: development of clustered eye-specific patterns in a novel target. J. Neurosci. 17, 2040–2055 9045732 10.1523/JNEUROSCI.17-06-02040.1997 PMC6793748

[B5] Annese J. Pitiot A. Dinov I. D. Toga A. W. (2004). A myelo-architectonic method for the structural classification of cortical areas. Neuroimage 21, 15–26 10.1016/j.neuroimage.2003.08.024 14741638

[B6] Anwander A. Tittgemeyer M. von Cramon D. Y. Friederici A. D. Knosche T. R. (2007). Connectivity-based parcellation of Broca's area. Cereb. Cortex 17, 816–825 10.1093/cercor/bhk034 16707738

[B7] Arthurs O. J. Johansen-Berg H. Matthews P. M. Boniface S. J. (2004). Attention differentially modulates the coupling of fMRI BOLD and evoked potential signal amplitudes in the human somatosensory cortex. Exp. Brain Res. 157, 269–274 10.1007/s00221-003-1827-4 15221172

[B8] Assaf Y. Basser P. J. (2005). Composite hindered and restricted model of diffusion (CHARMED) MR imaging of the human brain. Neuroimage 27, 48–58 10.1016/j.neuroimage.2005.03.042 15979342

[B9] Bach D. R. Behrens T. E. Garrido L. Weiskopf N. Dolan R. J. (2011). Deep and superficial amygdala nuclei projections revealed *in vivo* by probabilistic tractography. J. Neurosci. 31, 618–623 10.1523/JNEUROSCI.2744-10.2011 21228170 PMC3059574

[B10] Barbas H. (1986). Pattern in the laminar origin of corticocortical connections. J. Comp. Neurol. 252, 415–422 10.1002/cne.902520310 3793985

[B11] Barbas H. Rempel-Clower N. (1997). Cortical structure predicts the pattern of corticocortical connections. Cereb. Cortex 7, 635–616 9373019 10.1093/cercor/7.7.635

[B12] Barnard S. T. Pothen A. Simon H. (1995). A spectral algorithm for envelope reduction of sparse matrices. Numer. Linear Algebra Appl. 2, 317–334

[B13] Barnes K. A. Nelson S. M. Cohen A. L. Power J. D. Coalson R. S. Miezin F. M. Vogel A. C. Dubis J. W. Church J. A. Petersen S. E. Schlaggar B. L. (2011). Parcellation in left lateral parietal cortex is similar in adults and children. Cereb. Cortex 22, 1148–1158 10.1093/cercor/bhr189 21810781 PMC3328346

[B14] Barrick T. R. Lawes I. N. Mackay C. E. Clark C. A. (2007). White matter pathway asymmetry underlies functional lateralization. Cereb. Cortex 17, 591–598 10.1093/cercor/bhk004 16627859

[B15] Basser P. J. Pajevic S. Pierpaoli C. Duda J. Aldroubi A. (2000). *In vivo* fiber tractography using DT-MRI data. Magn. Reson. Med. 44, 625–632 10.1002/1522-2594(200010)44:4<625::AID-MRM17>3.0.CO;2-O 11025519

[B16] Beckmann C. F. DeLuca M. Devlin J. T. Smith S. M. (2005). Investigations into resting-state connectivity using independent component analysis. Philos. Trans. R. Soc. Lond. B Biol. Sci. 360, 1001–1013 10.1098/rstb.2005.1634 16087444 PMC1854918

[B17] Beckmann M. Johansen-Berg H. Rushworth M. F. S. (2009). Connectivity-based parcellation of human cingulate cortex and its relation to functional specialization. J. Neurosci. 29, 1175–1190 10.1523/JNEUROSCI.3328-08.2009 19176826 PMC6665147

[B18] Behrens T. E. J. Johansen-Berg H. (2005). Relating connectional architecture to grey matter function using diffusion imaging. Philos. Trans. R. Soc. Lond. B Biol. Sci. 360, 903–911 10.1098/rstb.2005.1640 16087435 PMC1854924

[B19] Behrens T. E. J. Johansen-Berg H. Jbabdi S. Rushworth M. F. S. Woolrich M. W. (2007). Probabilistic diffusion tractography with multiple fiber orientations: what can we gain? Neuroimage 34, 144–155 10.1016/j.neuroimage.2006.09.018 17070705 PMC7116582

[B20] Behrens T. E. J. Johansen-Berg H. Woolrich M. W. Smith S. M. Wheeler-Kingshott C. A. M. Boulby P. A. Barker G. J. Sillery E. L. Sheehan K. Ciccarelli O. Thompson A. J. Brady J. M. Matthews P. M. (2003a). Non-invasive mapping of connections between human thalamus and cortex using diffusion imaging. Nat. Neurosci. 6, 750–757 10.1038/nn1075 12808459

[B21] Behrens T. E. J. Woolrich M. W. Jenkinson M. Johansen-Berg H. Nunes R. G. Clare S. Matthews P. M. Brady J. M. Smith S. M. (2003b). Characterization and propagation of uncertainty in diffusion-weighted MR imaging. Magn. Reson. Med. 50, 1077–1088 10.1002/mrm.10609 14587019

[B22] Belmalih A. Borra E. Contini M. Gerbella M. Rozzi S. Luppino G. (2009). Multimodal architectonic subdivision of the rostral part (area F5) of the macaque ventral premotor cortex. J. Comp. Neurol. 512, 183–217 10.1002/cne.21892 19003790

[B23] Berman J. I. Chung S. W. Mukherjee P. Hess C. P. Han E. T. Henry R. G. (2008). Probabilistic streamline q-ball tractography using the residual bootstrap. Neuroimage 39, 215–222 10.1016/j.neuroimage.2007.08.021 17911030

[B24] Birn R. M. Diamond J. B. Smith M. A. Bandettini P. A. (2006). Separating respiratory-variation-related fluctuations from neuronal-activity-related fluctuations in fMRI. Neuroimage 31, 1536–1548 10.1016/j.neuroimage.2006.02.048 16632379

[B25] Birn R. M. Murphy K. Bandettini P. A. (2008a). The effect of respiration variations on independent component analysis results of resting state functional connectivity. Hum. Brain Mapp. 29, 740–750 10.1002/hbm.20577 18438886 PMC2715870

[B26] Birn R. M. Smith M. A. Jones T. B. Bandettini P. A. (2008b). The respiration response function: the temporal dynamics of fMRI signal fluctuations related to changes in respiration. Neuroimage 40, 644–654 10.1016/j.neuroimage.2008.09.029 18234517 PMC2533266

[B27] Biswal B. B. Mennes M. Zuo X.-N. Gohel S. Kelly C. Smith S. M. Beckmann C. F. Adelstein J. S. Buckner R. L. Colcombe S. Dogonowski A.-M. Ernst M. Fair D. Hampson M. Hoptman M. J. Hyde J. S. Kiviniemi V. J. Kotter R. Li S.-J. Lin C.-P. Lowe M. J. Mackay C. Madden D. J. Madsen K. H. Margulies D. S. Mayberg H. S. McMahon K. Monk C. S. Mostofsky S. H. Nagel B. J. Pekar J. J. Peltier S. J. Petersen S. E. Riedl V. Rombouts S. A. R. B. Rypma B. Schlaggar B. L. Schmidt S. Seidler R. D. Siegle G. J. Sorg C. Teng G.-J. Veijola J. Villringer A. Walter M. Wang L. Weng X.-C. Whitfield-Gabrieli S. Williamson P. Windischberger C. Zang Y.-F. Zhang H.-Y. Castellanos F. X. Milham M. M. (2010). Toward discovery science of human brain function. Proc. Natl. Acad. Sci. U.S.A. 107, 4734–4739 10.1073/pnas.0911855107 20176931 PMC2842060

[B28] Biswal B. Yetkin F. Z. Haughton V. M. Hyde J. S. (1995). Functional connectivity in the motor cortex of resting human brain using echo-planar MRI. Magn. Reson. Med. 34, 537–541 8524021 10.1002/mrm.1910340409

[B29] Blatt G. J. Pandya D. N. Rosene D. L. (2003). Parcellation of cortical afferents to three distinct sectors in the parahippocampal gyrus of the rhesus monkey: an anatomical and neurophysiological study. J. Comp. Neurol. 466, 161–179 10.1002/cne.10866 14528446

[B30] Bock N. A. Hashim E. Kocharyan A. Silva A. C. (2011). Visualising myeloarchitecture with magnetic resonance imaging in primates. Ann. N.Y. Acad. Sci. 1225, E171–E181 10.1111/j.1749-6632.2011.06000.x 21599695 PMC5027904

[B31] Bridge H. Clare S. Jenkinson M. Jezzard P. Parker A. J. Matthews P. M. (2005). Independent anatomical and functional measures of the V1/V2 boundary in human visual cortex. J. Vis. 5, 93–102 10.1167/5.2.1 15831070

[B32] Brodmann K. (1909). Localisation in the Cerebral Cortex. New York, NY: Springer

[B33] Broser P. Vargha-Khadem F. Clark C. A. (2011). Robust subdivision of the thalamus in children based on probability distribution functions calculated from probabilistic tractography. Neuroimage 57, 403–415 10.1016/j.neuroimage.2011.04.054 21570472

[B34] Buckner R. L. (2010). Human functional connectivity: new tools, unresolved questions. Proc. Natl. Acad. Sci. U.S.A. 107, 10769–10770 10.1073/pnas.1005987107 20547869 PMC2890705

[B35] Buckner R. L. Vincent J. L. (2007). Unrest at rest: default activity and spontaneous network correlations. Neuroimage 37, 1091–1096 10.1016/j.neuroimage.2007.01.010 17368915

[B36] Buckner R. L. Krienen F. M. Castellanos A. Diaz J. C. Yeo B. T. T. (2011). The organization of the human cerebellum estimated by intrinsic functional connectivity. J. Neurophysiol. 106, 2322–2345 10.1152/jn.00339.2011 21795627 PMC3214121

[B37] Buckner R. L. Sepulcre J. Talukdar T. Krienen F. M. Liu H. Hedden T. Andrews-Hanna J. R. Sperling R. A. Johnson K. A. (2009). Cortical hubs revealed by intrinsic functional connectivity: mapping, assessment of stability, and relation to Alzheimer's disease. J. Neurosci. 29, 1860–1873 10.1523/JNEUROSCI.5062-08.2009 19211893 PMC2750039

[B38] Bullmore E. T. Bassett D. S. (2011). Brain graphs: graphical models of the human brain connectome. Ann. Rev. Clin. Psychol. 7, 113–140 10.1146/annurev-clinpsy-040510-143934 21128784

[B39] Bullmore E. Sporns O. (2009). Complex brain networks: graph theoretical analysis of structural and functional systems. Nat. Rev. Neurosci. 10, 186–198 10.1038/nrn2575 19190637

[B40] Carmichael S. T. Price J. L. (1994). Architectonic subdivision of the orbital and medial prefrontal cortex in the macaque monkey. J. Comp. Neurol. 346, 366–402 10.1002/cne.10609 7527805

[B41] Caspers S. Eickhoff S. B. Geyer S. Scheperjans F. Mohlberg H. Zilles K. Amunts K. (2008). The human inferior parietal lobule in stereotaxic space. Brain Struct. Funct. 212, 481–495 10.1007/s00429-008-0195-z 18651173

[B42] Caspers S. Geyer S. Schleicher A. Mohlberg H. Amunts K. Zilles K. (2006). The human inferior parietal cortex: cytoarchitectonic parcellation and interindividual variability. Neuroimage 33, 430–448 10.1016/j.neuroimage.2006.06.054 16949304

[B43] Catani M. Allin M. P. G. Husain M. Pugliese L. Mesulam M. M. Murray R. M. Jones D. K. (2007). Symmetries in human brain language pathways correlate with verbal recall. Proc. Natl. Acad. Sci. U.S.A. 104, 17163–17168 10.1073/pnas.0702116104 17939998 PMC2040413

[B44] Catani M. Jones D. K. ffytche D. H. (2005). Perisylvian language networks of the human brain. Ann. Neurol. 57, 8–16 10.1002/ana.20319 15597383

[B45] Catani M. Thiebaut de Schotten M. (2008). A diffusion tensor imaging tractography atlas for virtual *in vivo* dissections. Cortex 44, 1105–1132 10.1016/j.cortex.2008.05.004 18619589

[B46] Cauda F. Costa T. Torta D. M. E. Sacco K. D'Agata F. Duca S. Geminiani G. Fox P. T. Vercelli A. (2012). Meta-analytic clustering of the insular cortex: characterising the meta-analytic connectivity of the insula when involved in active tasks. Neuroimage 62, 343–355 10.1016/j.neuroimage.2012.04.012 22521480 PMC4782788

[B47] Cauda F. D'Agata F. Sacco K. Duca S. Geminiani G. Vercelli A. (2011). Functional connectivity of the insula in the resting brain. Neuroimage 55, 8–23 10.1016/j.neuroimage.2010.11.049 21111053

[B48] Cavada C. Goldman-Rakic P. S. (1989). Posterior parietal cortex in rhesus monkey: I. Parcellation of areas based on distinctive limbic and sensory corticocortical connections. J. Comp. Neurol. 287, 393–421 10.1002/cne.902870402 2477405

[B49] Cerliani L. Thomas R. M. Jbabdi S. Siero J. C. W. Nanetti L. Crippa A. Gazzola V. D'Arceuil H. Keysers C. (2012). Probabilistic tractography recovers a rostrocaudal trajectory of connectivity variability in the human insular cortex. Hum. Brain Mapp. 33, 2005–2034 10.1002/hbm.21338 21761507 PMC3443376

[B50] Chang C. Cunningham J. P. Glover G. H. (2009). Influence of heart rate on the BOLD signal: the cardiac response function. Neuroimage 44, 857–869 10.1016/j.neuroimage.2008.09.029 18951982 PMC2677820

[B51] Chang L. J. Yarkoni T. Khaw M. W. Sanfrey A. G. (2012). Decoding the role of the insula in human cognition: functional parcellation and large-scale reverse inference. Cereb. Cortex. [Epub ahead of print]. 10.1093/cercor/bhs065 22437053 PMC3563343

[B52] Chuang K. H. Chen J. H. (2001). IMPACT: Image-based physiological artifacts estimation and correction technique for functional MRI. Magn. Reson. Med. 46, 344–353 10.1002/mrm.1197 11477639

[B53] Chung S. W. Lu Y. Henry R. G. (2006). Comparison of bootstrap approaches for estimation of uncertainties of DTI parameters. Neuroimage 33, 531–541 10.1016/j.neuroimage.2006.07.001 16938472

[B54] Cloutman L. L. Binney R. J. Drakesmith M. Parker G. J. M. Lambon Ralph M. A. (2012). The variation of function across the human insula mirrors its patterns of structural connectivity: evidence from *in vivo* probabilistic tractography. Neuroimage 59, 3514–3521 10.1016/j.neuroimage.2011.11.016 22100771

[B55] Cohen A. L. Fair D. A. Dosenbach N. U. F. Miezin F. M. Dierker D. Van Essen D. C. Schlaggar B. L. Petersen S. E. (2008). Defining functional areas in individual human brains using resting functional connectivity MRI. Neuroimage 41, 45–57 10.1016/j.neuroimage.2008.01.066 18367410 PMC2705206

[B56] Cohen-Adad J. Polimeni J. R. Helmer K. G. Benner T. McNab J. A. Wald L. L. Rosen B. R. Mainero C. (2012). T2* mapping and B0 orientation-dependence at 7 T reveal cyto- and myeloarchitecture organization of the human cortex. Neuroimage 60, 1006–1014 10.1016/j.neuroimage.2012.01.053 22270354 PMC3442114

[B57] Cole D. M. Smith S. M. Beckmann C. F. (2010). Advances and pitfalls in the analysis and interpretation of resting-state fMRI data. Front. Syst. Neurosci. 4:8 10.3389/fnsys.2010.00008 20407579 PMC2854531

[B58] Conturo T. E. Lori N. F. Cull T. S. Akbudak E. Snyder A. Z. Shimony J. S. McKinstry R. C. Burton H. Raichle M. E. (1999). Tracking neuronal fiber pathways in the living human brain. Proc. Natl. Acad. Sci. U.S.A. 96, 10422–10427 10.1073/pnas.96.18.10422 10468624 PMC17904

[B59] Craddock R. C. James G. A. Holtzheimer P. E. Hu X. P. Mayberg H. S. (2012). A whole brain fMRI atlas generated via spatially constrained spectral clustering. Hum. Brain Mapp. 33, 1914–1928 10.1002/hbm.21333 21769991 PMC3838923

[B60] Crippa A. Cerliani L. Nanetti L. Roerdink J. B. T. M. (2011). Heuristics for connectivity-based brain parcellation of SMA/pre-SMA through force-directed graph layout. Neuroimage 54, 2176–2184 10.1016/j.neuroimage.2010.09.075 20933092

[B61] Cruz-Rizzolo R. J. De Lima M. A. X. Ervolino E. de Oliveira J. A. Casatti C. A. (2011). Cyto-, myelo- and chemoarchitecture of the prefrontal cortex of the Cebus monkey. BMC Neurosci. 12, 6 10.1186/1471-2202-12-6 21232115 PMC3030535

[B62] Damoiseaux J. S. Greicius M. D. (2009). Greater than the sum of its parts: a review of studies combining structural connectivity and resting-state functional connectivity. Brain Struct. Funct. 213, 525–533 10.1007/s00429-009-0208-6 19565262

[B63] Dargi F. Oghabian M. A. Ahmadian A. Zadeh H. S. Zarei M. Boroomand A. (2007). Modified fast marching tractography algorithm and its ability to detect fiber crossing. Conf. Proc. IEEE Eng. Med. Miol. Soc. 2007, 319–322 10.1109/IEMBS.2007.4352288 18001954

[B64] Deco G. Corbetta M. (2011). The dynamical balance of the brain at rest. Neuroscientist 17, 107–123 10.1177/1073858409354384 21196530 PMC4139497

[B65] Devlin J. T. Sillery E. L. Hall D. A. Hobden P. Behrens T. E. J. Nunes R. G. Clare S. Matthews P. M. Moore D. R. Johansen-Berg H. (2006). Reliable identification of the auditory thalamus using multi-modal structural analyses. Neuroimage 30, 1112–1120 10.1016/j.neuroimage.2005.11.025 16473021 PMC1458525

[B66] Di Martino A. Scheres A. Margulies D. S. Kelly A. M. C. Uddin L. Q. Shehzad Z. Biswal B. Walters J. R. Castellanos F. X. Milham M. P. (2008). Functional connectivity of human striatum: a resting state fMRI study. Cereb. Cortex 18, 2735–2747 10.1093/cercor/bhn041 18400794

[B67] Ding S.-L. Van Hoesen G. W. Cassell M. D. Poremba A. (2009). Parcellation of human temporal polar cortex: a combined analysis of multiple cytoarchitectonic, chemoarchitectonic, and pathological markers. J. Comp. Neurol. 514, 595–623 10.1002/cne.22053 19363802 PMC3665344

[B68] Draganski B. Kherif F. Kloppel S. Cook P. A. Alexander D. C. Parker G. J. M. Deichmann R. Ashburner J. Frackowiak R. S. J. (2008). Evidence for segregated and integrative connectivity patterns in the human basal ganglia. J. Neurosci. 28, 7143–7152 10.1523/JNEUROSCI.1486-08.2008 18614684 PMC6670486

[B69] Dyrby T. B. Sogaard L. V. Parker G. J. Alexander D. C. Lind N. M. Baare W. F. C. Hay-Schmidt A. Eriksen N. Pakkenberg B. Paulson O. B. Jelsing J. (2007). Validation of *in vitro* probabilistic tractography. Neuroimage 37, 1267–1277 10.1016/j.neuroimage.2007.06.022 17706434

[B70] Eickhoff S. B. Bzdok D. Laird A. R. Roski C. Caspers S. Zilles K. Fox P. T. (2011). Co-activation patterns distinguish cortical modules, their connectivity and functional differentiation. Neuroimage 57, 938–949 10.1016/j.neuroimage.2011.05.021 21609770 PMC3129435

[B71] Eickhoff S. B. Schleicher A. Scheperjans F. Palomero-Gallagher N. Zilles K. (2007). Analysis of neurotransmitter receptor distribution patterns in the cerebral cortex. Neuroimage 34, 1317–1330 10.1016/j.neuroimage.2006.11.016 17182260

[B72] Eickhoff S. B. Schleicher A. Zilles K. Amunts K. (2006). The human parietal operculum. I. Cytoarchitectonic mapping of subdivisions. Cereb. Cortex 16, 254–267 10.1093/cercor/bhi105 15888607

[B73] Eickhoff S. B. Stephan K. E. Mohlberg H. Grefkes C. Fink G. R. Amunts K. Zilles K. (2005a). A new SPM toolbox for combining probabilistic cytoarchitectonic maps and functional imaging data. Neuroimage 25, 1325–1335 10.1016/j.neuroimage.2004.12.034 15850749

[B74] Eickhoff S. Walters N. B. Schleicher A. Kril J. Egan G. F. Zilles K. Watson J. D. G. Amunts K. (2005b). High-resolution MRI reflects myeloarchitecture and cytoarchitecture of human cerebral cortex. Hum. Brain Mapp. 24, 206–215 10.1002/hbm.20082 15543596 PMC6871702

[B75] Elias W. J. Zheng Z. A. Domer P. Quigg M. Pouratian N. (2012). Validation of connectivity-based thalamic segmentation with direct electrophysiologic recordings from human sensory thalamus. Neuroimage 59, 2025–2034 10.1016/j.neuroimage.2011.10.049 22036683

[B76] Felleman D. J. Van Essen D. C. (1991). Distributed hierarchical processing in the primate cerebral cortex. Cereb. Cortex 1, 1–47 10.1016/j.neuroimage.2009.04.061 1822724

[B77] Fingelkurts A. A. Fingelkurts A. A. Kahkonen S. (2005). Functional connectivity in the brain – is it an elusive concept? Neurosci. Biobehav. Rev. 28, 827–836 10.1016/j.neubiorev.2004.10.009 15642624

[B78] Fischl B. Rajendran N. Busa E. Augustinack J. Hinds O. Yeo B. T. T. Mohlberg H. Amunts K. Zilles K. (2008). Cortical folding patterns and predicting cytoarchitecture. Cereb. Cortex 18, 1973–1980 10.1093/cercor/bhm225 18079129 PMC2474454

[B79] Ford A. McGregor K. M. Case K. Crosson B. White K. D. (2010). Structural connectivity of Broca's area and medial frontal cortex. Neuroimage 52, 1230–1237 10.1016/j.neuroimage.2010.05.018 20488246 PMC2910137

[B80] Fox M. D. Raichle M. E. (2007). Spontaneous fluctuations in brain activity observed with functional magnetic resonance imaging. Nat. Rev. Neurosci. 8, 700–711 10.1038/nrn2201 17704812

[B81] Fox M. D. Snyder A. Z. Vincent J. L. Corbetta M. Van Essen D. C. Raichle M. E. (2005). The human brain is intrinsically organized into dynamic, anticorrelated functional networks. Proc. Natl. Acad. Sci. U.S.A. 102, 9673–9678 10.1073/pnas.0504136102 15976020 PMC1157105

[B82] Frey S. Campbell J. S. W. Pike G. B. Petrides M. (2008). Dissociating the human language pathways with high angular resolution diffusion fiber tractography. J. Neurosci. 28, 11435–11444 10.1523/JNEUROSCI.2388-08.2008 18987180 PMC6671318

[B83] Friston K. J. Frith C. D. Liddle P. F. Frackowiak R. S. J. (1993). Functional connectivity: the principal-component analysis of large (PET) data sets. J. Cereb. Blood Flow Metab. 13, 5–14 10.1038/jcbfm.1993.4 8417010

[B84] Gallay D. S. Gallay M. N. Jeanmonod D. Rouiller E. M. Morel A. (2012). The insula of Reil revisited: multiarchitectonic organization in macaque monkeys. Cereb. Cortex 22, 175–190 10.1093/cercor/bhr104 21613468 PMC3236796

[B85] Gerbella M. Belmalih A. Borra E. Rozzi S. Luppino G. (2007). Multimodal architectonic subdivision of the caudal ventrolateral prefrontal cortex of the macaque monkey. Brain Struct. Funct. 212, 269–301 10.1007/s00429-007-0158-9 17899184

[B86] Gerbella M. Belmalih A. Borra E. Rozzi S. Luppino G. (2010). Cortical connections of the macaque caudal ventrolateral prefrontal areas 45A and 45B. Cereb. Cortex 20, 141–168 10.1111/j.1460-9568.2010.07390.x 19406905

[B87] Geyer S. Luppino G. Ekamp H. Zilles K. (2005). The macaque inferior parietal lobule: cytoarchitecture and distribution pattern of serotonin 5-HT1A binding sites. Anat. Embryol. (Berl.) 210, 353–362 10.1007/s00429-005-0026-4 16180022

[B88] Geyer S. Schleicher A. Zilles K. (1997). The somatosensory cortex of human: cytoarchitecture and regional distributions of receptor-binding sites. Neuroimage 6, 27–45 10.1006/nimg.1997.0271 9245653

[B89] Geyer S. Schleicher A. Zilles K. (1999). Areas 3a, 3b, and 1 of human primary somatosensory cortex. I. Microstructural organization and interindividual variability. Neuroimage 10, 63–83 10.1006/nimg.1999.0440 10385582

[B90] Geyer S. Schormann T. Mohlberg H. Zilles K. (2000). Areas 3a, 3b, and 1 of human primary somatosensory cortex. II. Spatial normalization to standard anatomical space. Neuroimage 11, 684–696 10.1006/nimg.2000.0548 10860796

[B91] Geyer S. Weiss M. Reimann K. Lohmann G. Turner R. (2011). Microstructural parcellation of the human cerebral cortex – from Brodmann's post-mortem map to *in vivo* mapping with high-field magnetic resonance imaging. Front. Hum. Neurosci. 5:19 10.3389/fnhum.2011.00019 21373360 PMC3044325

[B92] Glasser M. F. Rilling J. K. (2008). DTI tractography of the human brain's language pathways. Cereb. Cortex 18, 2471–2482 10.1093/cercor/bhn011 18281301

[B93] Glasser M. F. Van Essen D. C. (2011). Mapping human cortical areas *in vivo* based on myelin content as revealed by T1- and T2-weighted MRI. J. Neurosci. 31, 11597–11616 10.1523/JNEUROSCI.2180-11.2011 21832190 PMC3167149

[B94] Gorbach N. S. Schutte C. Melzer C. Goldau M. Sujazow O. Jitsev J. Douglas T. Tittgemeyer M. (2011). Hierarchical information-based clustering for connectivity-based cortex parcellation. Front. Neuroinform. 5:18 10.3389/fninf.2011.00018 21977015 PMC3178812

[B95] Gregoriou G. G. Borra E. Matelli M. Luppino G. (2006). Architectonic organization of the inferior parietal convexity of the macaque monkey. J. Comp. Neurol. 496, 422–451 10.1002/cne.20933 16566007

[B96] Greicius M. D. Supekar K. Menon V. Dougherty R. F. (2009). Resting-state functional connectivity reflects structural connectivity in the default mode network. Cereb. Cortex 19, 72–78 10.1093/cercor/bhn059 18403396 PMC2605172

[B97] Hagmann P. Jonasson L. Maeder P. Thiran J.-P. Wedeen V. J. Meuli R. (2006). Understanding diffusion MR imaging techniques: from scalar diffusion-weighted imaging to diffusion tensor imaging and beyond. Radiographics 26, S205–S223 10.1148/rg.26si065510 17050517

[B98] Haroon H. A. Morris D. M. Embleton K. V. Alexander D. C. Parker G. J. M. (2009). Using the model-based residual bootstrap to quantify uncertainty in fiber orientations from q-ball analysis. IEEE Trans. Med. Imaging 28, 535–550 10.1109/TMI.2008.2006528 19272997

[B99] Hartigan J. A. Wong M. A. (1979). A k-means clustering algorithm. J. R. Stat. Soc. 28, 100–108

[B100] Hilgetag C.-C. Grant S. (2000). Unformity, specificity and variability of corticocortical connectivity. Philos. Trans. R. Soc. Lond. B Biol. Sci. 355, 7–20 10.1098/rstb.2000.0546 10703041 PMC1692717

[B101] Hilgetag C. C. Grant S. (2010). Cytoarchitectural differences are a key determinant of laminar projection origins in the visual cortex. Neuroimage 51, 1006–1017 10.1016/j.neuroimage.2010.03.006 20211270

[B102] Hof P. R. Morrison J. H. (1995). Neurofilament protein defines regional patterns of cortical organization in the macaque monkey visual system: a quantitative immunohistochemical analysis. J. Comp. Neurol. 352, 161–186 10.1002/cne.903520202 7721988

[B103] Honey C. J. Sporns O. Cammoun L. Gigandet X. Thiran J. P. Meuil R. Hagmann P. (2009). Predicting human resting-state functional connectivity from structural connectivity. Proc. Natl. Acad. Sci. U.S.A. 106, 2035–2040 10.1073/pnas.0811168106 19188601 PMC2634800

[B104] Honey C. J. Thivierge J.-P. Sporns O. (2010). Can structure predict function in the human brain? Neuroimage 52, 766–776 10.1016/j.neuroimage.2010.01.071 20116438

[B105] Horng S. H. Sur M. (2006). Visual activity and cortical rewiring: activity-dependent plasticity of cortical networks. Prog. Brain Res. 157, 3–11 17167899 10.1016/s0079-6123(06)57001-3

[B106] Jacobson S. Marcus E. M. (2008). Neuroanatomy for the Neuroscientist. New York, NY: Springer

[B107] Jakab A. Molnar P. P. Bogner P. Beres M. Berenyi E. L. (2012). Connectivity-based parcellation reveal interhemispheric differences in the insula. Brain Topogr. 25, 264–271 10.1007/s10548-011-0205-y 22002490

[B108] Jbabdi S. Johansen-Berg H. (2011). Tractography: where do we go from here? Brain Connect. 1, 169–183 10.1089/brain.2011.0033 22433046 PMC3677805

[B109] Jbabdi S. Woolrich M. W. Behrens T. E. J. (2009). Multiple-subjects connectivity-based parcellation using hierarchical Dirichlet process mixture models. Neuroimage 44, 373–384 10.1016/j.neuroimage.2008.08.044 18845262

[B110] Jian B. Vemuri B. C. (2007). A unified computational framework for deconvolution to reconstruct multiple fibers from diffusion weighted MRI. IEEE Trans. Med. Imaging 26, 1464–1471 10.1109/TMI.2007.907552 18041262 PMC2572690

[B111] Johansen-Berg H. Behrens T. E. J. (2009). Diffusion MRI: From Quantitative Measurement to In-Vivo Neuroanatomy. Amsterdam, Netherlands: Elsevier

[B112] Johansen-Berg H. Behrens T. E. J. Robson M. D. Drobnjak I. Rushworth M. F. S. Brady J. M. Smith S. M. Higham D. J. Matthews P. M. (2004). Changes in connectivity profiles define functionally distinct regions in human medial frontal cortex. Proc. Natl. Acad. Sci. U.S.A. 101, 13335–13340 10.1073/pnas.0403743101 15340158 PMC516567

[B113] Johansen-Berg H. Behrens T. E. J. Sillery E. Ciccarelli O. Thompson A. J. Smith S. M. Matthews P. M. (2005). Functional-anatomical validation and individual variation of diffusion tractography-based segmentation of the human thalamus. Cereb. Cortex 15, 31–39 10.1093/cercor/bhh105 15238447

[B114] Johansen-Berg H. Matthews P. M. (2002). Attention to movement modulates activity in sensori-motor areas, including primary motor cortex. Exp. Brain Res. 142, 13–24 10.1007/s00221-001-0905-8 11797080

[B115] Johansen-Berg H. Rushworth M. F. S. (2009). Using diffusion imaging to study human connectional anatomy. Ann. Rev. Neurosci. 32, 75–94 10.1146/annurev.neuro.051508.135735 19400718

[B116] Johnson S. C. (1967). Hierarchical clustering schemes. Psychometrika 32, 241–254 5234703 10.1007/BF02289588

[B117] Jones D. K. (2008a). Studying connections in the living brain with diffusion MRI. Cortex 44, 936–952 10.1016/j.cortex.2008.05.002 18635164

[B118] Jones D. K. (2008b). Tractography gone wild: probabilistic fiber tracking using the wild bootstrap with diffusion tensor MRI. IEEE Trans. Med. Imaging 27, 1268–1274 10.1109/TMI.2008.922191 18779066

[B119] Jones D. K. (2010). Challenges and limitations of quantifying brain connectivity *in vivo* with diffusion MRI. Imaging Med. 2, 341–355

[B120] Jones D. K. Pierpaoli C. (2005). Confidence mapping in diffusion tensor magnetic resonance imaging tractography using a bootstrap approach. Magn. Reson. Med. 53, 1143–1149 10.1002/mrm.20466 15844149

[B121] Jones D. K. Simmons A. Williams S. C. R. Horsfield M. A. (1999). Non-invasive assessment of axonal fiber connectivity in the human brain via diffusion tensor MRI. Magn. Reson. Med. 42, 37–41 10.1002/(SICI)1522-2594(199907)42:1<37::AID-MRM7>3.0.CO;2-O 10398948

[B122] Jones E. G. Burton H. (1976). Areal differences in the laminar distribution of thalamic afferents in cortical fields of the insular, parietal and temporal regions of primates. J. Comp. Neurol. 168, 197–248 10.1002/cne.901680203 821974

[B123] Kahnt T. Chang L. J. Park S. Q. Heinzle J. Haynes J.-D. (2012). Connectivity-based parcellation of the human orbitofrontal cortex. J. Neurosci. 32, 6240–6250 10.1523/JNEUROSCI.0257-12.2012 22553030 PMC6622144

[B124] Karkar S. Faisan S. Thoraval L. Foucher J. R. (2009). A multi-level parcellation approach for brain functional connectivity analysis. Conf. Proc. IEEE Eng. Med. Biol. Soc. 2009, 3497–3500 10.1109/IEMBS.2009.5334578 19964995

[B125] Keidel J. L. Welbourne S. R. Lambon Ralph M. A. (2010). Solving the paradox of the equipotential and modular brain: a nuerocomputational model of stroke vs. slow-growing glioma. Neuropsychologia 48, 1716–1724 10.1016/j.neuropsychologia.2010.02.019 20188115

[B126] Kelly C. Biswal B. B. Craddock R. C. Castellanos F. X. Milham M. P. (2012a). Characterising variation in the functional connectome: promise and pitfalls. Trends Cogn. Sci. 16, 181–188 10.1002/(SICI)1097-0193(1998)6:3<160::AID-HBM5>3.0.CO;2-1 22341211 PMC3882689

[B127] Kelly C. Toro R. De Martino A. Lox C. L. Bellec P. Castellanos F. X. Milham M. P. (2012b). A convergent functional architecture of the insula emerges across imaging modalities. Neuroimage 61, 1129–1142 10.1016/j.neuroimage.2012.03.021 22440648 PMC3376229

[B128] Kelly C. Uddin L. Q. Shehzad Z. Margulies D. S. Castellanos F. X. Milham M. P. Petrides M. (2010). Broca's region: linking human brain functional connectivity data and non-human primate tracing anatomy studies. Eur. J. Neurosci. 32, 383–398 10.1111/j.1460-9568.2010.07279.x 20662902 PMC3111969

[B129] Kim J.-H. Lee J.-M. Jo H. J. Kim S. H. Lee J. H. Kim S. T. Seo S. W. Cox R. W. Na D. L. Kim S. I. Saad Z. S. (2010). Defining functional SMA and pre-SMA subregions in human MFC using resting state fMRI: Functional connectivity-based parcellation method. Neuroimage 49, 2375–2386 10.1016/j.neuroimage.2009.10.016 19837176 PMC2819173

[B130] Kiviniemi V. Kantola J. H. Jauhiainen J. Hyvarinen A. Tervonen O. (2003). Independent component analysis of nondeterministic fMRI signal sources. Neuroimage 19, 253–260 10.1016/S1053-8119(03)00097-1 12814576

[B131] Klein J. C. Behrens T. E. J. Robson M. D. Mackay C. E. Higham D. J. Johansen-berg H. (2007). Connectivity-based parcellation of human cortex using diffusion MRI: establishing reproducibility, validity and observer independence in BA 44/45 and SMA/pre-SMA. Neuroimage 34, 204–211 10.1016/j.neuroimage.2006.08.022 17023184

[B132] Knosche T. R. Tittgemeyer M. (2011). The role of long-range connectivity for the characterization of the functional-anatomical organization of the cortex. Front Syst. Neurosci. 5:58 10.3389/fnsys.2011.00058 21779237 PMC3133730

[B133] Koch M. A. Norris D. G. Hund-Georgiadis M. (2002). An investigation of functional and anatomical connectivity using magnetic resonance imaging. Neuroimage 16, 241–250 10.1006/nimg.2001.1052 11969331

[B134] Kotter R. Hilgetag C. C. Stephan K. E. (2001a). Connectional characteristics of areas in Walker's map of primate prefrontal cortex. Neurocomputing 38-40, 741–746

[B135] Kotter R. Stephan K. E. Palomero-Gallagher N. Geyer S. Schleicher A. Zilles K. (2001b). Multimodal characterization of cortical areas by multivariate analyses of receptor binding and connectivity data. Anat. Embryol. (Berl.) 204, 333–350 10.1007/s004290100199 11720237

[B136] Lambert C. Zrinzo L. Nagy Z. Lutti A. Hariz M. Foltynie T. Draganski B. Ashburner J. Frackowiak R. (2012). Confirmation of functional zones within the human subthalamic nucleus: Patterns of connectivity and sub-parcellation using diffusion weighted imaging. Neuroimage 60, 83–94 10.1016/j.neuroimage.2011.11.082 22173294 PMC3315017

[B137] Lawes I. N. C. Barrick T. R. Murugam V. Spierings N. Evans D. R. Song M. Clark C. A. (2008). Atlas-based segmentation of white matter tracts of the human brain using diffusion tensor tractography and comparison with classical dissection. Neuroimage 39, 62–79 10.1016/j.neuroimage.2007.06.041 17919935

[B138] Lazar M. Alexander A. L. (2005). Bootstrap white matter tractography (BOOT-TRAC). Neuroimage 24, 524–532 10.1016/j.neuroimage.2004.08.050 15627594

[B139] Le Bihan D. (2007). The “wet mind”: water and functional neuroimaging. Phys. Med. Biol. 52, R57–R90 10.1088/0031-9155/52/7/R02 17374909

[B140] Leh S. E. Ptito A. Chakravarty M. M. Strafella A. P. (2007). Fronto-striatal connections in the human brain: a probabilistic diffusion tractography study. Neurosci. Lett. 419, 113–118 10.1016/j.neulet.2007.04.049 17485168 PMC5114128

[B141] Lehericy S. Ducros M. Van de Moortele P.-F. Francois C. Thivard L. Poupon C. Swindale N. Ugurbil K. Kim D.-K. (2004). Diffusion tensor fiber tracking shows distinct corticostriatal circuits in humans. Ann. Neurol. 55, 522–529 10.1002/ana.20030 15048891

[B142] Lloyd S. P. (1982). Least squares quantization in PCM. IEEE Trans. Inf. Theory 28, 129–137

[B143] Macey P. M. Macey K. E. Kumar R. Harper R. M. (2004). A method for removal of global effects from fMRI time series. Neuroimage 22, 360–366 10.1016/j.neuroimage.2003.12.042 15110027

[B144] Majewska A. K. Sur M. (2006). Plasticity and specificity of cortical processing networks. Trends Neurosci. 29, 323–329 10.1016/j.tins.2006.04.002 16697057

[B145] Makris N. Kennedy D. N. McInerney S. Sorensen A. G. Wang. R. Caviness V. S. Pandya D. N. (2005). Segmentation of subcomponents within the superior longitudinal fascicle in humans: a quantitative, *in vivo*, DT-MRI study. Cereb. Cortex 15, 854–869 10.1093/cercor/bhh186 15590909

[B146] Makris N. Pandya D. N. (2009). The extreme capsule in humans and rethinking of the language circuitry. Brain Struct. Funct. 213, 343–358 10.1007/s00429-008-0199-8 19104833 PMC3777634

[B147] Margulies D. S. Bottger J. Long X. Lv Y. Kelly C. Schafer A. Goldhahn D. Abbushi A. Milham M. P. Lohmann G. Villringer A. (2010). Resting developments: a review of fMRI post-processing methodologies for spontaneous brain activity. MAGMA 23, 289–307 10.1007/s10334-010-0228-5 20972883

[B148] Margulies D. S. Kelly A. M. C. Uddin L. Q. Biswal B. B. Castellanos F. X. Milham M. P. (2007). Mapping the functional connectivity of anterior cingulate cortex. Neuroimage 37, 579–588 10.1016/j.neuroimage.2007.05.019 17604651

[B149] Margulies D. S. Vincent J. L. Kelly C. Lohmann G. Uddin L. Q. Biswal B. B. Villringer A. Castellanos F. X. Milham M. P. Petrides M. (2009). Precuneus shares intrinsic functional architecture in humans and monkeys. Proc. Natl. Acad. Sci. U.S.A. 106, 20069–20074 10.1073/pnas.0905314106 19903877 PMC2775700

[B150] Mars R. B. Jbabdi S. Sallet J. O'Reilly J. X. Croxson P. L. Olivier E. Noonan M. P. Bergmann C. Mitchell A. S. Baxter M. G. Behrens T. E. J. Johansen-Berg H. Tomassini V. Miller K. L. Rushworth M. F. S. (2011). Diffusion-weighted imaging tractography-based parcellation of the human parietal cortex and comparison with human and macaque resting-state functional connectivity. J. Neurosci. 31, 4087–4100 10.1523/JNEUROSCI.5102-10.2011 21411650 PMC3091022

[B151] Mars R. B. Sallet J. Schuffelgen U. Jbabdi S. Toni I. Rushworth M. F. S. (2012). Connectivity-based subdivisions of the human right “temporoparietal junction area”: evidence for different areas participating in different cortical networks. Cereb. Cortex 22, 1894–1903 10.1093/cercor/bhr268 21955921

[B152] McKeown M. J. Makeig S. Brown G. G. Jung T.-P. Kindermann S. S. Bell A. J. Sejnowski T. J. (1998). Analysis of fMRI data by blind separation into independent spatial components. Hum. Brain Mapp. 6, 160–188 9673671 10.1002/(SICI)1097-0193(1998)6:3<160::AID-HBM5>3.0.CO;2-1 PMC6873377

[B153] Medalla M. Barbas H. (2006). Diversity of laminar connections linking periarcuate and lateral intraparietal areas depends on cortical structure. Eur. J. Neurosci. 23, 161–179 10.1111/j.1460-9568.2005.04522.x 16420426

[B154] Meindl T. Teipel S. Elmouden R. Mueller S. Koch W. Dietrich O. Coates U. Reiser M. Glaser C. (2010). Test-retest reproducibility of the default-mode network in healthy individuals. Hum. Brain Mapp. 31, 237–246 10.1002/hbm.20860 19621371 PMC6871144

[B155] Menke R. A. Jbabdi S. Miller K. L. Matthews P. M. Zarei M. (2010). Connectivity-based segmentation of the substantia nigra in human and its implications in Parkinson's disease. Neuroimage 52, 1175–1180 20677376 10.1016/j.neuroimage.2010.05.086

[B156] Mennes M. Kelly C. Colcombe S. Castellanos F. X. Milham M. P. (2012). The extrinsic and intrinsic functional architectures of the human brain are not equivalent. Cereb. Cortex. [Epub ahead of print]. 10.1093/cercor/bhs010 22298730 PMC3513960

[B157] Mesulam M. M. (2006). Foreword, in Fiber Pathways of the Brain, eds Schmahmann J. D. Pandya D. N. (New York, NY: Oxford University Press), ix–x

[B158] Mesulam M. M. Mufson E. J. (1982). Insula of the old world monkey. I: Architectonics in the insulo-orbito-temporal component of the paralimbic brain. J. Comp. Neurol. 212, 1–22 10.1002/cne.902120102 7174905

[B159] Mori S. (2007). Introduction to Diffusion Tensor Imaging. Amsterdam, Netherlands: Elsevier

[B160] Mori S. Crain B. J. Chacko V. P. Van Zijl P. C. (1999). Three-dimensional tracking of axonal projections in the brain by magnetic resonance imaging. Ann. Neurol. 45, 265–269 9989633 10.1002/1531-8249(199902)45:2<265::aid-ana21>3.0.co;2-3

[B161] Morosan P. Rademacher J. Schleicher A. Amunts K. Schormann T. Zilles K. (2001). Human primary auditory cortex: cytoarchitectonic subdivisions and mapping into a spatial reference system. Neuroimage 13, 684–701 10.1006/nimg.2000.0715 11305897

[B162] Morosan P. Schleicher A. Amunts K. Zilles K. (2005). Multimodal architectonic mapping of human superior temporal gyrus. Anat. Embryol. (Berl.) 210, 401–406 10.1007/s00429-005-0029-1 16170539

[B163] Mukherjee P. Berman J. I. Chung S. W. Hess C. P. Henry R. G. (2008a). Diffusion tensor MR imaging and fiber tractography: theoretical underpinnings. Am. J. Neuroradiol. 29, 632–641 10.3174/ajnr.A1051 18339720 PMC7978191

[B164] Mukherjee P. Chung S. W. Berman J. I. Hess C. P. Henry R. G. (2008b). Diffusion tensor MR imaging and fiber tractography: technical considerations. Am. J. Neuroradiol. 29, 843–852 10.3174/ajnr.A1052 18339719 PMC8128579

[B165] Murphy K. Birn R. M. Handwerker D. A. Jones T. B. Bandettini P. A. (2009). The impact of global signal regression on resting state correlations: are anti-correlated networks introduced? Neuroimage 44, 893–905 10.1016/j.neuroimage.2008.09.036 18976716 PMC2750906

[B166] Nanetti L. Cerliani L. Gazzola V. Renken R. Keysers C. (2009). Group analyses of connectivity-based cortical parcellation using repeated k-means clustering. Neuroimage 47, 1666–1677 10.1016/j.neuroimage.2009.06.014 19524682

[B167] Nelson S. M. Cohen A. L. Power J. D. Wig G. S. Mienin F. M. Wheeler M. E. Velanova K. Donaldson D. I. Phillips J. S. Schlaggar B. L. Petersen S. E. (2010). A parcellation scheme for human left lateral parietal cortex. Neuron 67, 156–170 10.1016/j.neuron.2010.05.025 20624599 PMC2913443

[B168] O'Muircheartaigh J. Vollmar C. Traynor C. Barker G. J. Kumari V. Symms M. R. Thompson P. Duncan J. S. Koepp M. J. Richardson M. P. (2011). Clustering probabilistic tractograms using independent component analysis applied to the thalamus. Neuroimage 54, 2020–2032 10.1016/j.neuroimage.2010.09.054 20884353 PMC3032893

[B169] Ongur D. Ferry A. T. Price J. L. (2003). Architectonic subdivision of the human orbital and medial prefrontal cortex. J. Comp. Neurol. 460, 425–449 10.1002/cne.10609 12692859

[B170] Osechinskiy S. Kruggel F. (2009). Quantitative comparison of high-resolution MRI and myelin-stained histology of the human cerebral cortex. Conf. Proc. IEEE Eng. Med. Biol. Soc. 2009, 85–89 10.1109/IEMBS.2009.5334695 19964920

[B171] Padberg J. Seltzer B. Cusick C. G. (2003). Architectonics and cortical connections of the upper bank of the superior temporal sulcus in the rhesus monkey: an analysis in the tangential plane. J. Comp. Neurol. 467, 418–434 10.1002/cne.10932 14608603

[B172] Pallas S. L. (2001). Intrinsic and extrinsic factors that shape neocortical specification. Trends Neurosci. 24, 417–423 10.1016/S0166-2236(00)01853-1 11410273

[B173] Parker G. J. M. Alexander D. C. (2003). Probabilistic Monte Carlo based mapping of cerebral connections utilizing whole-brain crossing fiber information. Inf. Process. Med. Imaging 18, 684–695 15344498 10.1007/978-3-540-45087-0_57

[B174] Parker G. J. M. Alexander D. C. (2005). Probabilistic anatomical connectivity derived from the microscopic persistence angular structure of cerebral tissue. Philos. Trans. R. Soc. Lond. B Biol. Sci. 360, 893–902 10.1098/rstb.2005.1639 16087434 PMC1854923

[B175] Parker G. J. M. Haroon H. A. Wheeler-Kingshott C. A. M. (2003). A framework for a streamline-based probabilistic index of connectivity PICo using a structural interpretation of MRI diffusion measurements. J. Magn. Reson. Imaging 18, 242–254 10.1002/jmri.10350 12884338

[B176] Parker G. J. M. Luzzi S. Alexander D. C. Wheeler-Kingshott C. A. M. Ciccarelli O. Lambon Ralph M. A. (2005). Lateralization of ventral and dorsal auditory-language pathways in the human brain. Neuroimage 24, 656–666 10.1016/j.neuroimage.2004.08.047 15652301

[B177] Parker G. J. M. Stephan K. E. Barker G. J. Rowe J. B. MacManus D. G. Wheeler-Kingshott C. A. M. Ciccarelli O. Passingham R. E. Spinks R. L. Lemon R. N. Turner R. (2002). Initial demonstration of *in vivo* tracing of axonal projections in the macaque brain and comparison with the human brain using diffusion tensor imaging and fast marching tractography. Neuroimage 15, 797–809 10.1006/nimg.2001.0994 11906221

[B178] Passingham R. E. Stephan K. E. Kotter R. (2002). The anatomical basis of functional localization in the cortex. Nat. Rev. Neurosci. 3, 606–616 10.1038/nrn893 12154362

[B179] Perrin M. Cointepas Y. Cachia A. Poupon C. Thirion B. Riviere D. Cathier P. El Kouby V. Constantinesco A. Le Bihan D. Mangin J.-F. (2008). Connectivity-based parcellation of the cortical mantle using q-ball diffusion imaging. Int. J. Biomed. Imaging 2008, 368406 10.1155/2008/368406 18401457 PMC2288697

[B180] Petrides M. Pandya D. N. (2009). Distinct parietal and temporal pathways to the homologues of Broca's area in the monkey. PLoS Biol. 7:e1000170 10.1371/journal.pbio.1000170 19668354 PMC2714989

[B181] Purves D. Augustine G. J. Fitzpatrick D. Hall W. C. LaMantia A.-S. McNamara J. O. Williams S. M. (2004). Neuroscience, 3rd Edn Sunderland, MA: Sinauer Associates

[B182] Ragsdale C. W. Grove E. A. (2001). Patterning the mammalian cerebral cortex. Curr. Opin. Neurobiol. 11, 50–58 10.1016/S0959-4388(00)00173-2 11179872

[B183] Raichle M. E. (2011). The restless brain. Brain Connect. 1, 3–12 10.1089/brain.2011.0019 22432951 PMC3621343

[B184] Rakic P. Suner I. Williams R. W. (1991). A novel cytoarchitectonic area induced experimentally within the primate visual cortex. Proc. Natl. Acad. Sci. U.S.A. 88, 2083–2087 2006147 10.1073/pnas.88.6.2083 PMC51173

[B185] Rao S. M. Binder J. R. Bandettini P. A. Hammeke T. A. Yetkin F. Z. Jesmanowicz A. Lisk L. M. Morris G. L. Mueller W. M. Estkowski L. D. Wong E. C. Haughton V. M. Hyde J. S. (1993). Functional magnetic resonance imaging of complex human movements. Neurology 43, 2311–2318 8232948 10.1212/wnl.43.11.2311

[B186] Rempel-Clower N. L. Barbas H. (2000). The laminar pattern of connections between prefrontal and anterior temporal cortices in the rhesus monkey is related to cortical structure and function. Cereb. Cortex 10, 851–865 10982746 10.1093/cercor/10.9.851

[B187] Rivier F. Clarke S. (1997). Cytochrome oxidase, acetylcholinesterase, and NADPH-diaphorase staining in human supratemporal and insular cortex: evidence for multiple auditory areas. Neuroimage 6, 288–304 10.1006/nimg.1997.0304 9417972

[B188] Roca P. Riviere D. Guevara P. Poupon C. Mangin J.-F. (2009). Tractography-based parcellation of the cortex using a spatially-informed dimension reduction of the connectivity matrix. Med. Image Comput. Comput. Assist. Interv. 12, 935–942 20426078 10.1007/978-3-642-04268-3_115

[B189] Roca P. Tucholka A. Riviere D. Guevara P. Poupon C. Mangin J.-F. (2010). Inter-subject connectivity based parcellation of a patch of cerebral cortex. Med. Image Comput. Comput. Assist. Interv. 13, 347–354 20879334 10.1007/978-3-642-15745-5_43

[B190] Rockland K. S. Pandya D. N. (1979). Laminar origins and terminations of cortical connections of the occipital lobe in the rhesus monkey. Brain Res. 179, 3–20 10.1016/0006-8993(79)90485-2 116716

[B191] Roe A. W. Pallas S. L. Kwon Y. H. Sur M. (1992). Visual projections routed to the auditory pathway in ferrets: receptive fields of visual neurons in primary auditory cortex. J. Neurosci. 12, 3651–3664 1527604 10.1523/JNEUROSCI.12-09-03651.1992 PMC6575717

[B192] Roy A. K. Shehzad Z. Margulies D. S. Kelly A. M. C. Uddin L. Q. Gotimer K. Biswal B. B. Castellanos F. X. Milham M. P. (2009). Functional connectivity of the human amygdala using resting state fMRI. Neuroimage 45, 614–626 10.1016/j.neuroimage.2008.11.030 19110061 PMC2735022

[B193] Rozzi S. Calzavara R. Belmalih A. Borra E. Gregoriou G. G. Matelli M. Luppino G. (2006). Cortical connections of the inferior parietal cortical convexity of the macaque monkey. Cereb. Cortex 16, 1389–1417 10.1093/cercor/bhj076 16306322

[B194] Rubinov M. Sporns O. (2010). Complex network measures of brain connectivity: uses and interpretations. Neuroimage 52, 1059–1069 10.1016/j.neuroimage.2009.10.003 19819337

[B195] Rykhlevskaia E. Gratton G. Fabiani M. (2008). Combining structural and functional neuroimaging data for studying brain connectivity: a review. Psychophysiology 45, 173–187 10.1111/j.1469-8986.2007.00621.x 17995910

[B196] Saur D. Kreher B. W. Schnell S. Kummerer D. Kellmeyer P. Vry M.-S. Umarova R. Musso M. Glauche V. Abel S. Huber W. Rijntjes M. Hennig J. Weiller C. (2008). Ventral and dorsal pathways for language. Proc. Natl. Acad. Sci. U.S.A. 105, 18035–18040 10.1073/pnas.0805234105 19004769 PMC2584675

[B197] Saur D. Schelter B. Schness S. Kratochvil D. Kupper H. Kellmeyer P. Kummerer D. Kloppel S. Glauche V. Lange R. Mader W. Feess D. Timmer J. Weiller C. (2010). Combining functional and anatomical connectivity reveals brain networks for auditory language comprehension. Neuroimage 49, 3187–3197 10.1016/j.neuroimage.2009.11.009 19913624

[B198] Scheperjans F. Grefkes C. Palomero-Gallagher N. Schleicher A. Zilles K. (2005a). Subdivisions of human parietal area 5 revealed by quantitative receptor autoradiography: a parietal region between motor, somatosensory, and cingulate cortical areas. Neuroimage 25, 975–992 10.1016/j.neuroimage.2004.12.017 15808998

[B199] Scheperjans F. Palomero-Gallagher N. Grefkes C. Schleicher A. Zilles K. (2005b). Transmitter receptors reveal segregation of cortical areas in the human superior parietal cortex: relations to visual and somatosensory regions. Neuroimage 28, 362–379 10.1016/j.neuroimage.2005.06.028 16054841

[B200] Scheperjans F. Hermann K. Eickhoff S. B. Amunts K. Schleicher A. Zilles K. (2008). Observer-independent cytoarchitectonic mapping of the human superior parietal cortex. Cereb. Cortex 18, 846–867 10.1093/cercor/bhm116 17644831

[B201] Schleicher A. Amunts K. Geyer S. Morosan P. Zilles K. (1999). Observer-independent method for microstructural parcellation of cerebral cortex: a quantitative approach to cytoarchitectonics. Neuroimage 9, 165–177 10.1006/nimg.1998.0385 9918738

[B202] Schleicher A. Palomero-Gallagher N. Morosan P. Eickhoff S. B. Kowalski T. de Vos K. Amunts K. Zilles K. (2005). Quantitative architectural analysis: a new approach to cortical mapping. Anat. Embryol. (Berl.) 210, 373–386 10.1007/s00429-005-0028-2 16249867

[B203] Schubotz R. I. Anwander A. Knosche T. R. von Cramon D. Y. Tittgemeyer M. (2010). Anatomical and functional parcellation of the human lateral premotor cortex. Neuroimage 50, 396–408 10.1016/j.neuroimage.2009.12.069 20035880

[B204] Sharma J. Angelucci A. Sur M. (2000). Induction of visual orientation modules in auditory cortex. Nature 404, 841–847 10.1038/35009043 10786784

[B205] Shehzad Z. Kelly A. M. C. Reiss P. Gee D. G. Gotimer K. Uddin L. Q. Lee S. H. Margulies D. S. Roy A. K. Biswal B. B. Petkova E. Castellanos F. X. Milham M. P. (2009). The resting brain: unconstrained yet reliable. Cereb. Cortex 19, 2209–2229 10.1093/cercor/bhn256 19221144 PMC3896030

[B206] Sims K. S. Williams R. S. (1990). The human amygdaloid complex: a cytologic and histochemical atlas using nissl, myelin, acetylcholinesterase and nicotinamide adenine dinucleotide phosphate diaphorase staining. Neuroscience 36, 449–472 10.1016/0306-4522(90)90440-F 1699167

[B207] Skudlarski P. Jagannathan K. Calhoun V. D. Hampson M. Skudlarska B. A. Pearlson G. (2008). Measuring brain connectivity: Diffusion tensor imaging validates resting state temporal correlations. Neuroimage 43, 554–561 10.1016/j.neuroimage.2008.07.063 18771736 PMC4361080

[B208] Solano-Castiella E. Anwander A. Lohmann G. Weiss M. Docherty C. Geyer S. Reimer E. Friederici A. D. Turner R. (2010). Diffusion tensor imaging segments the human amygdala *in vivo*. Neuroimage 49, 2958–2965 10.1016/j.neuroimage.2009.11.027 19931398

[B209] Sporns O. (2011). The human connectome: a complex network. Ann. N.Y. Acad. Sci. 1224, 109–125 10.1111/j.1749-6632.2010.05888.x 21251014

[B210] Sporns O. Tononi G. Kotter R. (2005). The human connectome: a structural description of the human brain. PLoS Comput. Biol. 1:e42 10.1371/journal.pcbi.0010042 16201007 PMC1239902

[B211] Stam C. J. (2010). Characterization of anatomical and functional connectivity in the brain: a complex networks perspective. Int. J. Psychophysiol. 77, 186–194 10.1016/j.ijpsycho.2010.06.024 20598763

[B212] Stejskal E. O. Tanner J. E. (1965). Spin diffusion measurements: spin echoes in the presence of a time dependent field gradient. J. Chem. Phys. 42, 288–292

[B213] Sur M. Garraghty P. E. Roe A. W. (1988). Experimentally induced visual projections into auditory thalamus and cortex. Science 242, 1437–1441 2462279 10.1126/science.2462279

[B214] Toga A. W. Thompson P. M. Mori S. Amunts K. Zilles K. (2006). Towards multimodal atlases of the human brain. Nat. Rev. Neurosci. 7, 952–966 10.1038/nrn2012 17115077 PMC3113553

[B215] Tomassini V. Jbabdi S. Klein J. C. Behrens T. E. J. Pozzilli C. Matthews P. M. Rushworth M. F. S. Johansen-Berg H. (2007). Diffusion-weighted imaging tractography-based parcellation of the human lateral premotor cortex identifies dorsal and ventral subregions with anatomical and functional specializations. J. Neurosci. 27, 10259–10269 10.1523/JNEUROSCI.2144-07.2007 17881532 PMC6672665

[B216] Tournier J.-D. Yeh C.-H. Calamante F. Cho K.-H. Connelly A. Lin C.-P. (2008). Resolving crossing fibers using constrained spherical deconvolution: validation using diffusion-weighted imaging phantom data. Neuroimage 42, 617–625 10.1016/j.neuroimage.2008.05.002 18583153

[B217] Traynor C. Heckemann R. A. Hammers A. O'Muircheartaigh J. Crum W. R. Barker G. J. Richardson M. P. (2010). Reproducibility of thalamic segmentation based on probabilistic tractography. Neuroimage 52, 69–85 10.1016/j.neuroimage.2010.04.024 20398772

[B218] Turken A. U. Dronkers N. F. (2011). The neural architecture of the language comprehension network: converging evidence from lesion and connectivity analyses. Front. Syst. Neurosci. 5:1 10.3389/fnsys.2011.00001 21347218 PMC3039157

[B219] Tyszka J. M. Kennedy D. P. Adolphs R. Paul L. K. (2011). Intact bilateral resting-state networks in the absence of the corpus callosum. J. Neurosci. 31, 15154–15162 10.1523/JNEUROSCI.1453-11.2011 22016549 PMC3221732

[B220] Uddin L. Q. Mooshagian E. Zaidel E. Scheres A. Margulies D. S. Kelly A. M. C. Shehzad Z. Adelstein J. S. Castellanos F. X. Biswal B. B. Milham M. P. (2008). Residual functional connectivity in the split-brain revealed with resting-state functional MRI. Neuroreport 19, 703–709 10.1097/WNR.0b013e3282fb8203 18418243 PMC3640406

[B221] Van Buuren M. Gladwin T. E. Zandbelt B. B. van den Heuvel M. Ramsey N. F. Kahn R. S. Vink M. (2009). Cardiorespiratory effects on default-mode network activity as measured with fMRI. Hum. Brain Mapp. 30, 3031–3042 10.1002/hbm.20729 19180557 PMC6871216

[B222] van den Heuvel M. P. Hulshoff Pol H. E. (2010). Exploring the brain network: a review on resting-state fMRI functional connectivity. Eur. Neuropsychopharmacol. 20, 519–534 10.1016/j.euroneuro.2010.03.008 20471808

[B223] Van Dijk K. R. A. Hedden T. Venkataraman A. Evans K. C. Lazar S. W. Buckner R. L. (2010). Intrinsic functional connectivity as a tool for human connectomics: theory, properties, and optimization. J. Neurophysiol. 103, 297–321 10.1152/jn.00783.2009 19889849 PMC2807224

[B224] Van Essen D. C. Maunsell J. H. R. (1983). Hierarchical organization and functional streams in the visual cortex. Trends Neurosci. 6, 370–375

[B225] Wallace M. N. Johnston P. W. Palmer A. R. (2002). Histochemical identification of cortical areas in the auditory region of the human brain. Exp. Brain Res. 143, 499–508 10.1007/s00221-002-1014-z 11914796

[B226] Walters N. B. Egan G. F. Kril J. J. Kean M. Waley P. Jenkinson M. Watson J. D. G. (2003). *In vivo* identification of human cortical areas using high-resolution MRI: an approach to cerebral structure-function correlation. Proc. Natl. Acad. Sci. U.S.A. 100, 2981–2986 10.1073/pnas.0437896100 12601170 PMC151452

[B227] Wedeen V. J. Wang R. P. Schmahmann J. D. Benner T. Tseng W. Y. I. Dai G. Pandya D. N. Hagmann P. D'Arceuil H. de Crespigny A. J. (2008). Diffusion spectrum magnetic resonance imaging (DSI) tractography of crossing fibers. Neuroimage 41, 1267–1277 10.1016/j.neuroimage.2008.03.036 18495497

[B228] Wise R. Ide K. Poulin M. J. Tracey I. (2004). Resting fluctuations in arterial carbon dioxide induce significant low frequency variations in BOLD signal. Neuroimage 21, 1652–1664 10.1016/j.neuroimage.2003.11.025 15050588

[B229] Yeo B. T. T. Krienen F. M. Sepulcre J. Sabuncu M. R. Lashkari D. Hollinshead M. Roffman J. L. Smoller J. W. Zollei L. Polimeni J. R. Fischl B. Liu H. Buckner R. L. (2011). The organization of the human cerebral cortex estimated by intrinsic functional connectivity. J. Neurophysiol. 106, 1125–1165 10.1152/jn.00338.2011 21653723 PMC3174820

[B230] Yu C. Zhou Y. Liu Y. Jiang T. Dong H. Zhang Y. Walter M. (2011). Functional segregation of the human cingulate cortex is confirmed by functional connectivity based neuroanatomical parcellation. Neuroimage 54, 2571–2581 10.1016/j.neuroimage.2010.11.018 21073967

[B231] Zarahn E. Aguirre G. K. D'Esposito M. (1997). Empirical analyses of BOLD fMRI statistics. I. Spatially unsmoothed data collected under null-hypothesis conditions. Neuroimage 5, 179–197 10.1006/nimg.1997.0263 9345548

[B232] Zhang D. Snyder A. Z. Fox M. D. Sansbury M. W. Shimony J. S. Raichle M. E. (2008). Intrinsic functional relations between human cerebral cortex and thalamus. J. Neurophysiol. 100, 1740–1748 10.1152/jn.90463.2008 18701759 PMC2576214

[B233] Zhang D. Snyder A. Z. Shimony J. S. Fox M. D. Raichle M. E. (2010). Noninvasive functional and structural connectivity mapping of the human thalamocortical system. Cereb. Cortex 20, 1187–1194 10.1093/cercor/bhp182 19729393 PMC2852505

[B234] Zilles K. Amunts K. (2010). Centenary of Brodmann's map – conception and fate. Nat. Rev. Neurosci. 11, 139–145 10.1038/nrn2776 20046193

[B235] Zilles K. Palomero-Gallagher N. Grefkes C. Scheperjans F. Boy C. Amunts K. Schleicher A. (2002a). Architectonics of the human cerebral cortex and transmitter receptor fingerprints: Reconciling functional neuroanatomy and neurochemistry. Eur. Neuropsychopharmacol. 12, 587–599 12468022 10.1016/s0924-977x(02)00108-6

[B236] Zilles K. Schleicher A. Palomero-Gallagher N. Amunts K. (2002b). Quantitative analysis of cyto- and receptor architecture of the human brain, in Brain Mapping: The Methods, 2nd Edn, eds Toga A. Mazziotta J. (San Diego, CA: Academic Press), 573–602

[B237] Zilles K. Palomero-Gallagher N. (2001). Cyto-, myelo-, and receptor architectonics of the human parietal cortex. Neuroimage 14, S8–S20 10.1006/nimg.2001.0823 11373127

[B238] Zilles K. Palomero-Gallagher N. Schleicher A. (2004). Transmitter receptors and functional anatomy of the cerebral cortex. J. Anat. 205, 417–432 10.1111/j.0021-8782.2004.00357.x 15610391 PMC1571403

[B239] Zilles K. Schlaug G. Matelli M. Luppino G. Schleicher A. Qu M. Dabringhaus A. Seitz R. Roland P. E. (1995). Mapping of human and macaque sensorimotor areas by integrating architectonic, transmitter receptor, MRI and PET data. J. Anat. 187, 515–537 8586553 PMC1167457

[B240] Zuo X. N. Di Martino A. Kelly C. Shehzad Z. E. Gee D. G. Klein D. F. Castellanos F. X. Biswal B. B. Milham M. P. (2010a). The oscillating brain: complex and reliable. Neuroimage 49, 1432–1445 10.1016/j.neuroimage.2009.09.037 19782143 PMC2856476

[B241] Zuo X. N. Kelly C. Adelstein J. S. Klein D. F. Castellanos F. X. Milham M. P. (2010b). Reliable intrinsic connectivity networks: test-retest evaluation using ICA and dual regression approach. Neuroimage 49, 2163–2177 10.1016/j.neuroimage.2009.10.080 19896537 PMC2877508

